# Plasticity in memristive devices for spiking neural networks

**DOI:** 10.3389/fnins.2015.00051

**Published:** 2015-03-02

**Authors:** Sylvain Saïghi, Christian G. Mayr, Teresa Serrano-Gotarredona, Heidemarie Schmidt, Gwendal Lecerf, Jean Tomas, Julie Grollier, Sören Boyn, Adrien F. Vincent, Damien Querlioz, Selina La Barbera, Fabien Alibart, Dominique Vuillaume, Olivier Bichler, Christian Gamrat, Bernabé Linares-Barranco

**Affiliations:** ^1^Laboratoire d'Intégration du Matériau au Système, UMR CNRS 5218, Université de BordeauxTalence, France; ^2^Institute of Neuroinformatics, University of Zurich and ETH ZurichZurich, Switzerland; ^3^Instituto de Microelectrónica de Sevilla, IMSE-CNM, Universidad de Sevilla and CSICSevilla, Spain; ^4^Faculty of Electrical Engineering and Information Technology, Technische Universität ChemnitzChemnitz, Germany; ^5^Unité Mixte de Physique CNRS/Thales, Palaiseau, France Associated to University Paris-SudOrsay, France; ^6^Institut d'Electronique Fondamentale, Université Paris-Sud, CNRSOrsay, France; ^7^Institut d'Electronique, Microelectronique et Nanotechnologies, UMR CNRS 8520Villeneuve d'Ascq, France; ^8^CEA, LIST, Saclay Nano-INNOV PC 172Gif sur Yvette, France

**Keywords:** memristive device, memristor, neuromorphic engineering, plasticity, hardware neural network

## Abstract

Memristive devices present a new device technology allowing for the realization of compact non-volatile memories. Some of them are already in the process of industrialization. Additionally, they exhibit complex multilevel and plastic behaviors, which make them good candidates for the implementation of artificial synapses in neuromorphic engineering. However, memristive effects rely on diverse physical mechanisms, and their plastic behaviors differ strongly from one technology to another. Here, we present measurements performed on different memristive devices and the opportunities that they provide. We show that they can be used to implement different learning rules whose properties emerge directly from device physics: real time or accelerated operation, deterministic or stochastic behavior, long term or short term plasticity. We then discuss how such devices might be integrated into a complete architecture. These results highlight that there is no unique way to exploit memristive devices in neuromorphic systems. Understanding and embracing device physics is the key for their optimal use.

## Introduction

In 1971, Leon Chua indicated the possible existence of a fourth basic electrical component (Chua, 1971). This component, the memristor, would complement those already known namely resistance, capacitor, and inductor, and offer new opportunities for system design (Chua and Kang, 1976). In particular, Chua proposed to use memristors or similar memristive devices to fabricate synapses and neurons following the Hodgkin–Huxley formalism. From this theoretical work, several publications have cited the memristive phenomenon without naming it as such and without linking it to Chua's theory (Upadhyaya and Chandra, 1995; Lau et al., 2004; Waser and Aono, 2007; Wu et al., 2007; Pershin and Di Ventra, 2008). HP labs were the first to recognize a device as a memristor in 2008 (Strukov et al., 2008), and they highlighted both the technology and its possible applications.

In parallel, the designers of the neuromorphic community worked hard on achieving CMOS neurons to reach electrical energy consumption of the order of picojoule per spike (Wijekoon and Dudek, 2008; Livi and Indiveri, 2009; Rangan et al., 2010; Merolla et al., 2011; Joubert et al., 2012). However, if the neuron implementation still have to face important challenges to match the neurons density and functionality required for neuromorphic circuits, the most abundant element in a neural network is the synapse. Consequently, most of the efforts have been concentrated on achieving high density memories with embedded synaptic functionalities (i.e., synaptic plasticity) in a single component. To become functional, the realization of a plastic synapse requires three parts: (i) synaptic weight storage, (ii) circuit for updating this weight depending on the network activity, and (iii) circuit for information transmission between two neurons. The neuromorphic community has developed a strong interest in memristive devices because these nanodevices and the associated integration strategies offer potential solutions to realize these three functions.

Resistive Random Access Memory (ReRAM) technologies in its broad sense have been developed for pure memory applications but can fall into the memristive system classification (Baek et al., 2004; Lee et al., 2008; Wong et al., 2012). These different technologies are mostly used in binary mode and are at the stage of industrialization and commercialization (e.g., ReRAM from Panasonic and Samsung) with high endurance, low energy, and high integration capability performances (Kawahara et al., 2012; Liu et al., 2013). Such performances can be an interesting platform for the implementation of synaptic weight storage (even in binary mode) if integration strategies and specific architectures are developed in order to offer a suitable solution to the large access required between neuron (i.e., computing node) and synapses (memory) inherent to parallel computing in neuromorphic circuits (and unsolved by Von Neumann architectures and associated bottleneck). In addition, their use in analog mode (or multilevel), is the subject of great attention and could be an effective solution for the implementation of synaptic functions.

**Figure 1:**
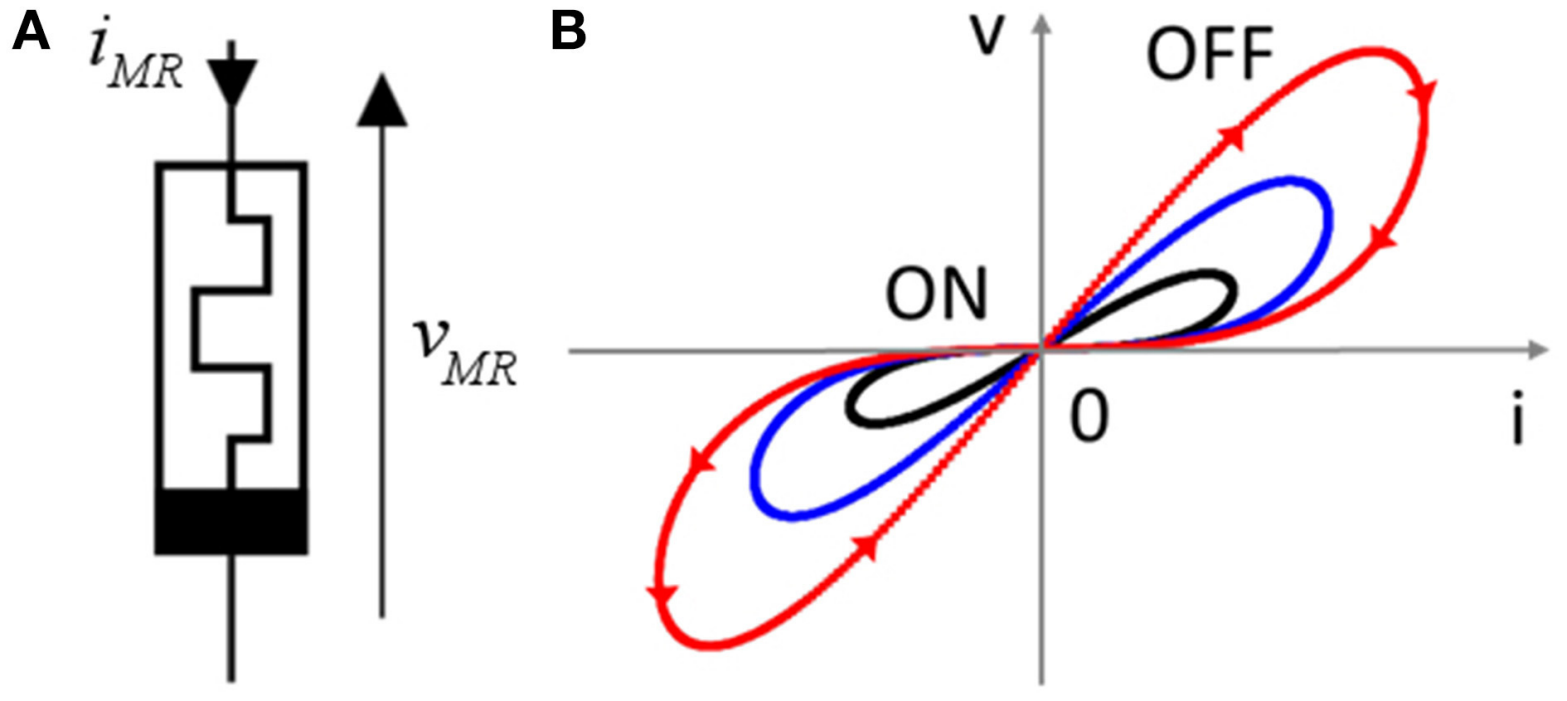
**(A)** Symbol of memristor; **(B)** characteristic transport features of memristors: pinched iv loops for different values of the maximum injected current.

**Figure 2:**
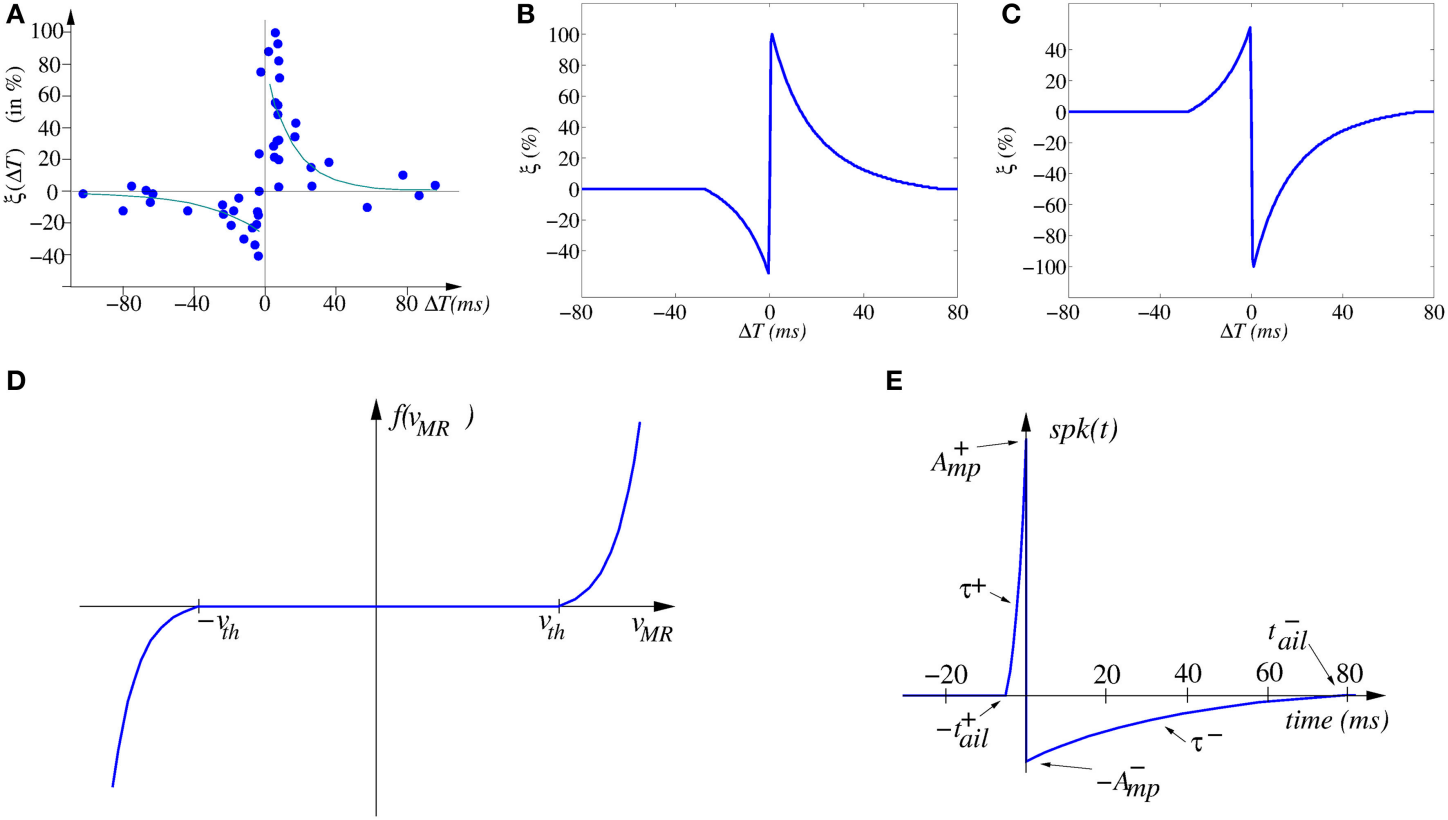
**(A)** Experimentally measured STDP function ξ_*i*_(**Δ***T*) on biological synapses (data from Bi and Poo, 1998, 2001). **(B)** Ideal STDP update function used in computational models of STDP synaptic learning. **(C)** Anti-STDP learning function for inhibitory STDP synapses. **(D)** Shape of memristor weight update function *f*(*v*_*MR*_). **(E)** Spike shape waveform.

Defining a memristor itself (see Figure 1A) can be debatable. Leon Chua now defines a memristor as any element that has an I(V) curve pinched at 0 V (Figure 1B) (Chua, 2014). This definition is widely used in the literature for characterizing devices, and in this paper we synonymously use the historic word memristor or the more generic “memristive device.” A general feature of memristive devices is to offer a non-volatile modification of its resistance (or conductance) as a function of the current (charge) or voltage (flux) driving the device. In particular, neuromorphic circuit designers prefer to think of memristors as resistive components that have the following properties: (i) the greater the electrical charge that has passed through the component, the more the resistance value decreases, (ii) the resistance value is stored in the element even after it is turned off. Moreover, this modification appears if the charge through the memristor goes over a “threshold” (Figure 2D).

Memristors can be realized using several technologies and we can categorize these technologies in four large families. The first includes anionic and cationic Red-Ox devices operating on Oxidation–Reduction principles. The second is phase-change memories (PCM), where resistive switching is connected with a physical phase change. Organic elements represent the third family. The fourth family finally comprises elements using purely electronic effects such as ferroelectric tunnel and spintronic memristors. These technologies possess different behaviors and therefore different fields of application. As part of this paper about synaptic plasticity, we also point out that these technologies will lead to different plastic behaviors and learning rules. These differences enrich the palette of possibilities for neuromorphic design. As Jeong et al. (2013), the purpose of this paper is not to present an exhaustive list of memristive technology and of their associated behavior, but rather to present the different forms of learning that have been observed. In our paper, all data about memristive devices have been measured by at least one of the co-authors.

If computing and memorization principles in neural networks are not completely understood, it is now widely recognized that learning in such systems is associated to synaptic weight modification that tends to reinforce or depress the strength of the connection between two neurons and grouped into the wide class of synaptic plasticity. The most popular description of learning was proposed by Hebb with the postulate “who fire together, wire together” (Hebb, 1949). In other words, two neurons presenting a correlated activity will tend to reinforce their synaptic connection. A first requirement is to define what we call neuron activity: two different approaches are commonly used, (i) rate coding strategies correspond to the definition of neuron activity as the mean firing rate estimated on a chosen time window while (ii) temporal coding corresponds to the assignment of neuron activity to a single spike event with a given time stamp with respect to the other spiking neurons considered in the network. Based on this different coding strategies, variations of Hebbian learning have been proposed such has Spike Rate Dependent Plasticity (SRDP) or the very popular Spike Timing Dependent Plasticity (STDP). In particular, STDP has attracted a large interest in the memristive device community because of its practical implementation based on overlapping pulses coming from the pre and post neurons. We present in Section STDP Learning Thanks to Overlapping Events theoretical elements that allow the understanding of the application of this basic learning algorithm. Starting from this ideal case, we present practical implementations of STDP in solid state devices and show how material constraint (i.e., switching mechanism, operating conditions, …) can be used to realize various form of STDP. Then we present two cases of “ferroelectric” memristors based on thin film semiconductor-metal-metaloxide compounds. These compounds were some of the first materials to be used as memristive synapses (see Kuzum et al., 2013 for a review). The first of our ferroelectric memristors is based on several 100 nm thick BiFeO_3_ films experiencing resistive switching in the Schottky barrier formed with one of the contacts. Specifically, the memristive effect in these devices is effected by a change of the depletion layer of the Schottky diode due to a non-volatile charge transfer similar to the “moving barrier” of TiO_2_. The second consists of ferroelectric tunnel junctions of very thin (~1 nm) BiFeO_3_ films in which tunneling resistance is linked to the polarization of the barrier. They differ radically by the time scales on which they operate and thus by the contexts in which they could be used. A third case based on spin-transfer torque magnetic tunnel junction is also presented in Section Spin-Transfer Torque Magnetic Tunnel Junction as a Stochastic Synapse. It presents a stochastic behavior in learning which is in some ways reminiscent of biological neural networks. In Section SRDP with Memristive Devices, we present different form of SRDP observed in biological synapses and of interest for spike rate coding strategies. We first show how Short Term Plasticity, corresponding to a temporary modification of the weight that tends to relax toward a resting state, can be used to implement rate dependent modification of the weight. A second example describes how Short Term/Long Term plasticity transitions can be reproduced by taking advantage of device stability characteristics. Before the conclusion, Section Toward Memristor-CMOS Architectures and Circuits opens the discussion on the characteristics of circuit architectures that will drive memristors following their electrical behavior.

## STDP learning thanks to overlapping events

### Theoretical principles

STDP is the ability of natural or artificial synapses to change their strength according to the precise timing of individual pre- and/or post-synaptic spikes (Gerstner et al., 1993, 1996; Markram et al., 1997; Bi and Poo, 1998, 2001; Zhang et al., 1998; Feldman, 2000; Mu and Poo, 2006; Cassenaer and Laurent, 2007; Jacob et al., 2007; Young, 2007; Finelli et al., 2008; Masquelier et al., 2008, 2009). A comprehensive overview of STDP and of its history can be found elsewhere (Sjöström and Gerstner, 2010). STDP learning in biology is inherently asynchronous and on-line, meaning that synaptic incremental update occurs while neurons and synapses transmit spikes and perform computations in parallel. Early proposals of this used artificial time-multiplexing to alternate continuously and synchronously between “performing” and “weight update” phases (Snider, 2008), thus requiring global system-wide synchronization. This can become a severe handicap when scaling up systems. Another option is a fully asynchronous implementation for memristor-based STDP where “performing” and “weight update” phases happen simultaneously in a natural manner, as in biology (Linares-Barranco and Serrano-Gotarredona, 2009a,b; Zamarreño-Ramos et al., 2011; Bichler et al., 2012b; Kuzum et al., 2012), and where there is no need for any global synchronization.

Figure 2A shows the change of synaptic strength (in percent) measured experimentally from biological synapses as function of relative timing Δ*T* = *t*_pos_ − *t*_pre_ between the arrival time *t*_*pre*_ of a pre-synaptic spike and the time *t*_*pos*_ of the generation of a post-synaptic spike. Although the data shows stochasticity, we can infer an underlying interpolated function ξ(Δ*T*) as shown in Figure 2B.

(1) $$\xi \left(\Delta T\right)=\{\begin{array}{c} a^{+}e^{-\frac{\Delta T}{\tau^{+}}}\;if\;\Delta T>0\\ -a^{-}e^{-\frac{\Delta T}{\tau^{-}}}\;if\;\Delta T<0 \end{array}$$

For a causal pre- to post-spike timing relation (Δ*T* > 0) the strength of the synapse is increased, while for an anti-causal relation (Δ*T* < 0) it is decreased. In the case of synapses with negative synaptic strength (as in some artificial realizations), the reversed version shown in Figure 2C can be used. Microchip CMOS circuit implementations of STDP rules that follow the description of Equation (1) have been reported (Indiveri et al., 2006), which result in about 30 transistors per plastic synapse, and thus may lead to high costs for their hardware realization. There is, overall, general thinking that STDP is very expensive to implement in conventional CMOS microchips (Fieres et al., 2008; Khan et al., 2008). However, it can be implemented with just one memristor per synapse if appropriate peripheral signal conditioning neurons are used in hybrid CMOS/memristor realizations.

For our purpose, we will consider a particular type of memristors, named voltage/flux driven memristor, which can be mathematically defined by.

(2) $$\begin{array}{c} i_{\;MR}=G\left(w,v_{\;MR}\right)v_{\;MR}\\ \dot{w}=f\left(v_{\;MR}\right) \end{array}$$

Memristor current and voltage are in general related through a non-linear conductance *G* (in the *i*_*MR*_ vs. *v*_*MR*_ plane), whose shape is tuned by parameter *w*. Most of the times, however, we may approximate the conductance as being totally linear *i*_*MR*_ = *G*(*w*)*v*_*MR*_, where the value of *w* is dependent on the history of *v*_*MR*_. Parameter *w* represents some structural property of the memristor. This parameter changes non-linearly as a function *f*( ) of the evolution of the memristor voltage *v*_*MR*_, so that the derivative of *w* is governed by the second equation in (Equation 2). A typical shape of this function is shown in Figure 2D, where a “dead zone” between two threshold voltages is present. While the memristor voltage is kept within this dead zone, parameter *w* will remain constant, and *G* will not change. But if the memristor voltage goes out of the dead zone, the (linear or non-linear conductance *G*) will change.

The STDP learning rule (as modeled by Equation 1) can, in theory, be implemented by (i) using a particular type of voltage/flux driven memristor (Jo et al., 2010), while (ii) providing appropriately shaped pre- and post-synaptic spikes available at both synapse (memristor) electrodes (Zamarreño-Ramos et al., 2011). For example, we can consider a pair of identical pre- and post-synaptic spikes with a shape resembling that of biological spikes (see Figure 2E), with an on-set duration |*t*^+^_*ail*_| and a tail of duration |*t*^−^_*ail*_|,

(3) $$spk\left(t\right)=\{\begin{array}{cc} A_{mp}^{+}\frac{e^{\frac{t}{\tau^{+}}}-e^{-\frac{t_{ail}^{+}}{\tau^{+}}}}{1-e^{-\frac{t_{ail}^{+}}{\tau^{+}}}} & if\;-t_{ail}^{+}<t<0\\ -A_{mp}^{-}\frac{e^{-\frac{t}{\tau^{-}}}-e^{-\frac{t_{ail}^{-}}{\tau^{-}}}}{1-e^{-\frac{t_{ail}^{-}}{\tau^{-}}}} & if\;0<t<t_{ail}^{-}\\ 0 & if\;otherwise \end{array}$$

**Figure 3:**
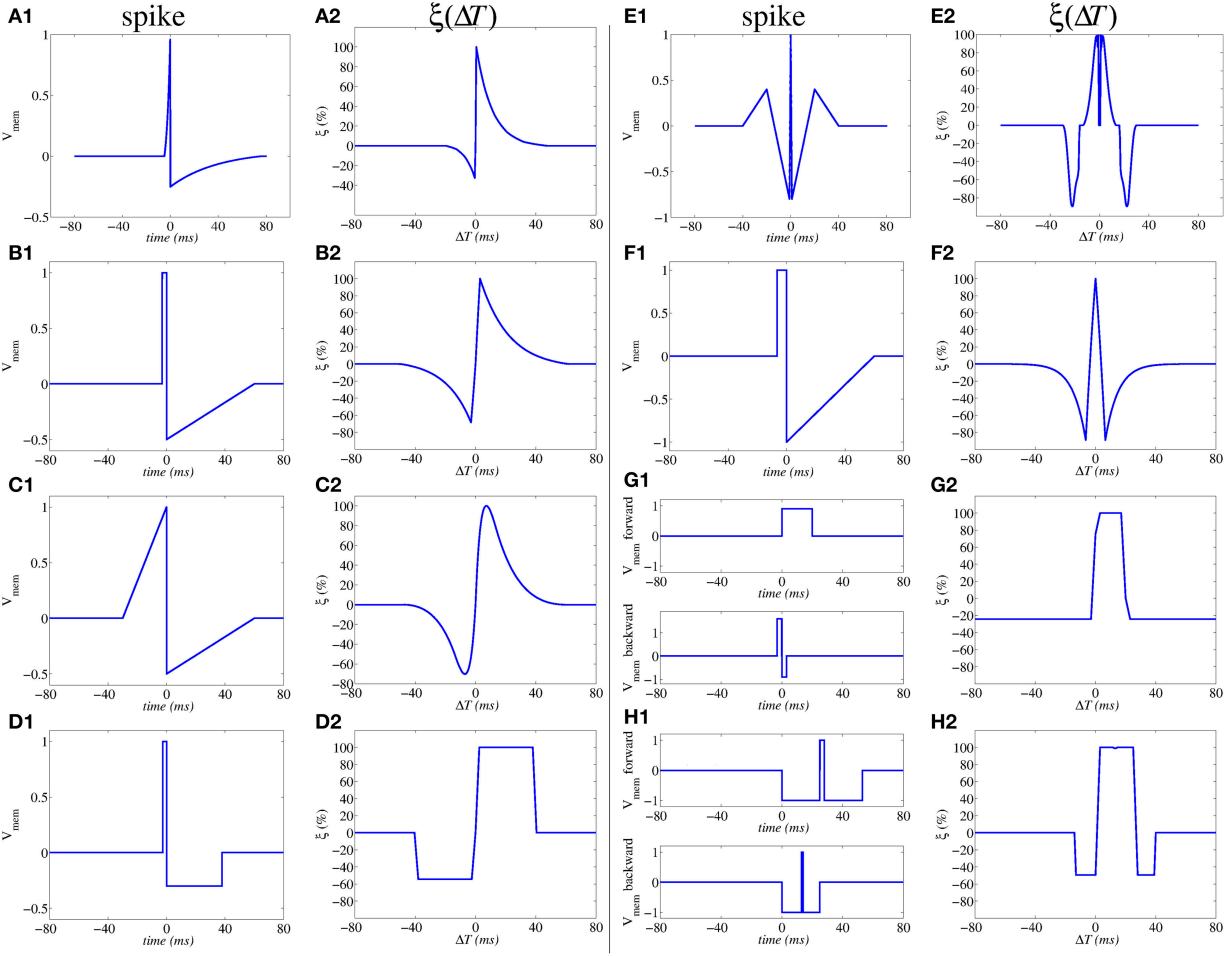
**Illustration of influence of action potential shapes on the resulting STDP memristor weight update function ξ(Δ*T*).** Memristor upper and lower thresholds are normalized to amplitudes ±1.0. From **(A1,A2)** to **(E1,E2)** the same spike waveform travels forward and backward. In **(F1,F2)** the forward and backward waveforms are the same but have opposite polarity. In **(G1,G2)** to **(H1,H2)** the forward and backward waveforms are different. In **(G1,G2)**, the positive pulse of the backward waveform exceeds amplitude +1.0, thus producing negative STDP update whenever there is a post-synaptic spike alone **(G2)**; otherwise if pre- and post-synaptic spikes happen within a given time window, there will be positive STDP update.

Under these circumstances, memristor voltage is *v*_*MR*_(*t*, Δ*t*) = α_*pos*_ *spk*(*t*) – α_*pre*_ *spk*(*t* + Δ*t*) and synaptic strength change can be computed as.

(4) $$\Delta w\left(\Delta T\right)=\int f\left(v_{MR}\left(t,\;\Delta T\right)\right)dt=\xi \left(\Delta T\right)$$

which has been shown to result in the same shape illustrated in Figure 2B (Zamarreño-Ramos et al., 2011). Furthermore, by reshaping the spike waveform, one can fine tune or completely alter the STDP learning function ξ(Δ*T*), as illustrated in Figure 3. This way, by building neurons with a given degree of shape programmability, it is possible to change the STDP learning function at will, depending on the application, or make it evolve in time as learning progresses.

**Figure 4:**
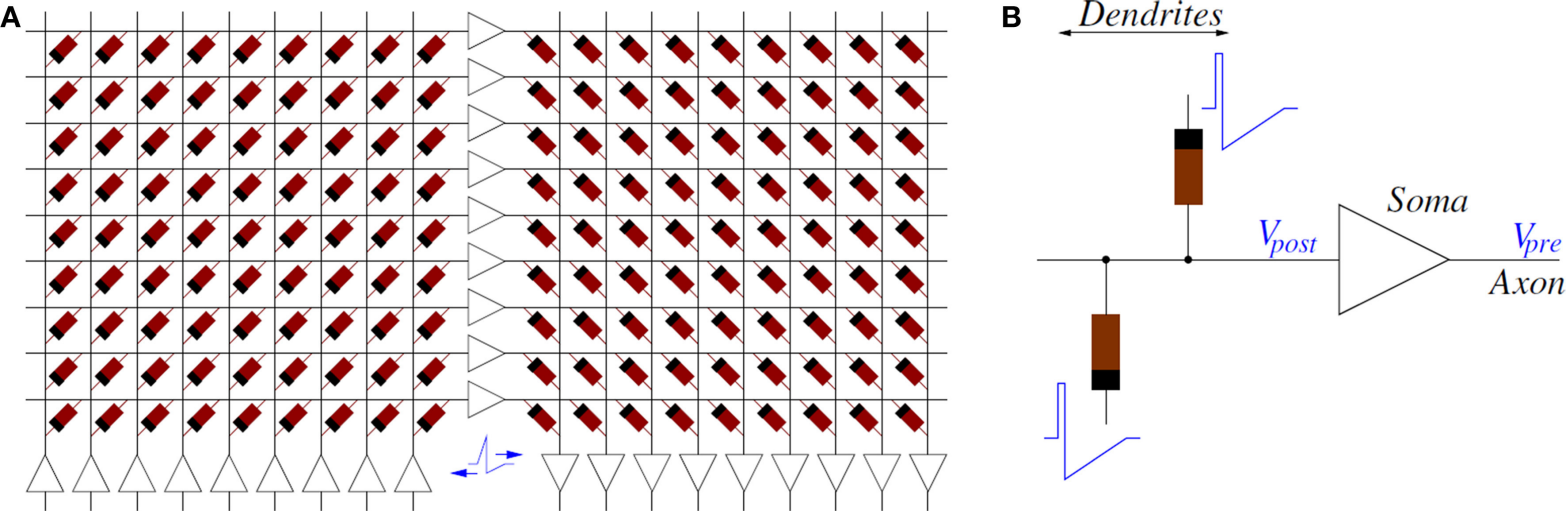
**(A)** Example of Memristors and CMOS neuron circuits arrangement for achieving STDP learning: feed-forward neural system with 3 layers of neurons and two fully connecting synapse crossbars. **(B)** Details of parts around one post-synaptic neuron. While a neuron is silent, it sets a constant DC voltage at its input (*V*_*post*_) and output (*V*_*pre*_) nodes. When a neuron is sending a spike, it sets a voltage spike at both nodes.

Figure 4A shows a way of interconnecting memristors and CMOS neurons for STDP learning. Triangles represent the neuron soma, the flat side indicating its input (dendrites) and the sharp side its output (axon). Dark rectangles are memristors, each representing one synaptic junction. Every neuron controls the voltage at its input (*V*_*post*_ in Figure 4B) and output (*V*_*pre*_ in Figure 4B) nodes. When the neuron is not spiking it forces a constant voltage at both nodes, while collecting through its input node the sum of input synaptic spike currents coming from the memristors, which contribute to changing the neuron internal state. When the neuron spikes, it sets a one-spike waveform at both input and output nodes. This way, they send their output spikes forward as pre-synaptic spikes for the destination synaptic memristors, but also backward to preceding synaptic memristors as post-synaptic spikes. Zamarreño et al. showed extensive simulations on these concepts, and how one can change from STDP to anti-STDP by switching polarities of spikes or memristors (Zamarreño-Ramos et al., 2011). For example, (Figures 3F1,F2) illustrate the case where forward and backward spikes have opposite polarities, resulting in a symmetric STDP update function ξ(Δ*T*). Figures 3G1,G2 illustrate an example where forward and backward spikes are different, with the backward spike such that its positive part exceeds the positive memristor threshold (*v*_*th*_ = 1.0). This produces LTD (long term depression) or negative STDP update whenever there is a post-synaptic spike sufficiently apart from a pre-synaptic one; and produces LTP (long term potentiation) if pre- and post-synaptic spikes happen within a given time window (Bichler et al., 2012a,b). Figures 2H1,H2 illustrate a similar STDP update behavior, except that the update (whether positive or negative) is restricted to a limited time window.

If the system is structured into neural layers (for example, Figure 4A shows a 3-neuron-layer system) with memristive synapses in between, then for each layer all pre-synaptic neurons should have the same forward spike shape and all post-synaptic neurons should have the same backward shape. This way, all memristive synapses between these two neural layers will have the same STDP function ξ(Δ*T*).

### Waveform-defined plasticity in ferroelectric resistive switching memristors

**Figure 5:**
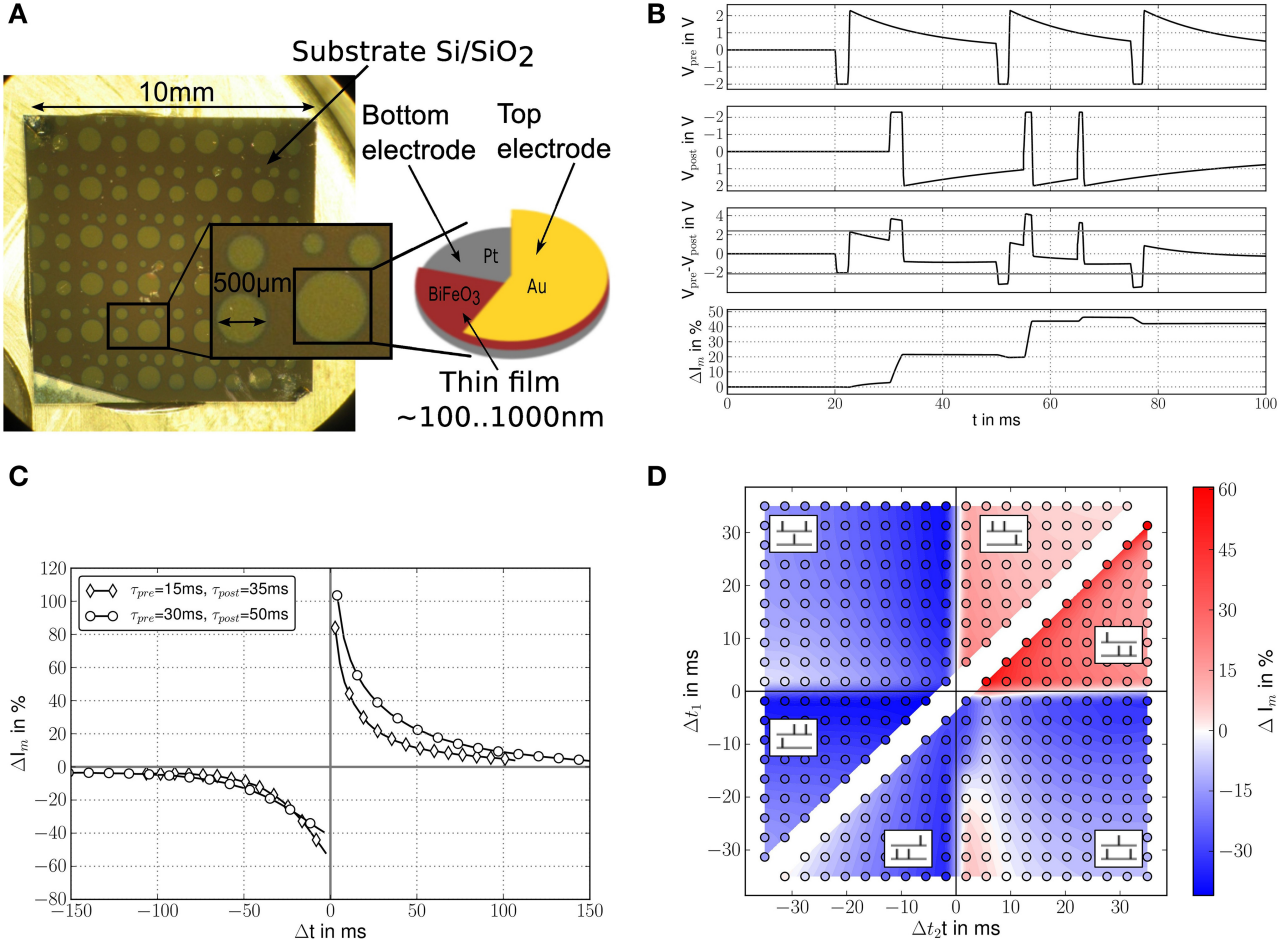
**(A)** Layout/processing of BiFeO3 devices used (Shuai et al., 2013); **(B)** driving voltage waveforms (from top to bottom): pre-synaptic waveform, post-synaptic waveform, resulting differential voltage across memristor and resulting memristance change shown as percentage change in current through the memristor for a fixed 2 V measurement voltage (Cederstroem et al., 2013); **(C)** measured STDP curves for two different STDP time window settings; time windows are adjusted via the time constants of the exponentials slopes of pre- and post-synaptic waveform, which changes the LTP respectively the LTD part of the STDP window; Weight change as change in current through the memristor; **(D)** measured spike triplet curves (Froemke and Dan, 2002), weight change as change in current through the memristor (Mayr et al., 2012).

In this section, we concentrate on an analysis of resistive switching BiFeO3 (BFO). Our BFO memristors are grown by pulsed laser deposition on Pt/Ti/SiO2/Si substrate with a circular Au top contact (Shuai et al., 2013), see Figure 5A. The BFO films have a thickness of some 100 nm. The top contact forms a Schottky diode, causing the created devices to show resistive switching with a rectifying behavior (Shuai et al., 2011). The devices exhibit a combination of voltage- and charge-driven behavior, and are consistent with the requirements of Section Theoretical Principles. When stepping DC voltages across the device, the resistance will follow an exponential curve (Mayr et al., 2012). The voltage level defines the converged resistance value, while the charge passed through the device defines the time frame until this converged value is achieved.

Resistive switching in BFO shows a number of characteristics which make it well-suited for use as a synapse. For instance, the dependence between voltage level and converged resistance makes the BFO devices conform closely to the ideal waveform-driven plasticity postulated in Figure 3, as plastic changes in the memristor closely follow the overlapping pre- and post-synaptic waveforms. Up to 8 bit analog resolution can be reliably programmed in the device (Shuai et al., 2013). Due to the Schottky diode, there is also high-ohmic region up to 1 V. Similar to the paradigm of Linn et al. (2010), this can be used in an array of BFO devices to define a voltage readout-region where only a single device in the array is active, eliminating the multiple sneak current paths that would otherwise severely limit practical array size (Flocke and Noll, 2007). While this characteristic potentially enables large crossbar arrays of BFO devices, defect density is on the order of 30% for an “open circuit” type failure, so a placement algorithm (Mayr et al., 2007) would have to be used in a memristive array to map around defect memristors.

The devices also experience a modification threshold at ca. 2 V, i.e., starting from the Schottky diode threshold at 1 V up to 2 V, the memristance can be measured by the current flow, but the charge inherent in this current does not change the memristance. If appropriate waveforms are chosen, the 2 V threshold extracts pre- and post-synaptic activity correlation as memristance change, as postulated in Section Theoretical Principles. All these voltages are broadly compatible with CMOS logic processes, in contrast to other material choices that need significantly higher voltages (Kuzum et al., 2013).

The waveforms in the upper two curves of Figure 5B are used as pre- respectively post-synaptic voltage. Those curves have not been shown in Figure 3; however their asymmetry is in the spirit of Figures 3G1,H1. These waveforms implement the plasticity model of Mayr et al. (2010), which allows for both rate- and spike-based plastic behavior. In the third curve of Figure 5B, which shows the resulting differential voltage across the memristor, the modification thresholds at about 2 V are marked. As can be seen, these are crucial in permitting modification only for true pre-post coincidences (such as at 30 ms), filtering out single pre- or post-synaptic events (such as at 20 ms). The resulting synaptic modification is shown in the last curve of Figure 5B, exhibiting a close match with the theoretical model (Mayr et al., 2010).

Measured STDP curves using this paradigm are shown in Figure 5C. With their exact reproduction of the waveform-defined exponential time window, they showcase the capability of BFO synapses for fine-grained analog weights. In most current memristive materials, the STDP curves deviate significantly more, and their time windows are primarily defined by the physical device characteristics, not the driving waveform (Alibart et al., 2012; Kuzum et al., 2013). In contrast, the voltage-memristance relationship of the BFO synapses lets them conform nicely to the waveform-defines-plasticity paradigm postulated in theory (Zamarreño-Ramos et al., 2011). Through this direct translation of the driving voltage waveforms into the plasticity shape, different time windows can be easily configured via the pre- and post-synaptic waveforms, as can be seen from the two sample curves in Figure 5C.

By introducing adaptation into the post-synaptic waveform, specifically an exponential dependence of the post-synaptic action potential duration on the inter-spike interval, the plasticity rule of Mayr and Partzsch (2010) is also able to reproduce triplet and rate plasticity (Froemke and Dan, 2002). When exploring the triplet paradigm with memristors, a faithful reproduction of biological triplet data can be seen (Figure 5D), due again to the excellent correlation between driving waveform and evoked memristive plasticity. The post-synaptic adaptation introduced for triplet plasticity can be observed in the different pulse widths in the second curve in Figure 5B (Noack et al., 2010).

Defining the plasticity entirely through the waveform can also be used to substantially speed up synapse behavior in BFO up to a 50 μs time scale (You et al., 2014). A switched capacitor system such as (Mayr et al., 2014b), if equipped with a scalable time base (Eisenreich et al., 2009), also offers the intriguing possibility of operating a high-density, CMOS-memristor hybrid neuromorphic system at varying timescales to accommodate different tasks, such as real-time interoperation with a visual sensor vs. offline, high-speed classification tasks where an accelerated timescale leads to faster classification.

### High-speed plasticity in ferroelectric tunnel memristors

“Purely electronic” memristors are nanodevices in which the resistance changes are obtained through electron mediated phenomena at interfaces. These memristors promise an increased endurance and reliability, since the material structure is preserved, as well as a faster switching speed.

**Figure 6:**
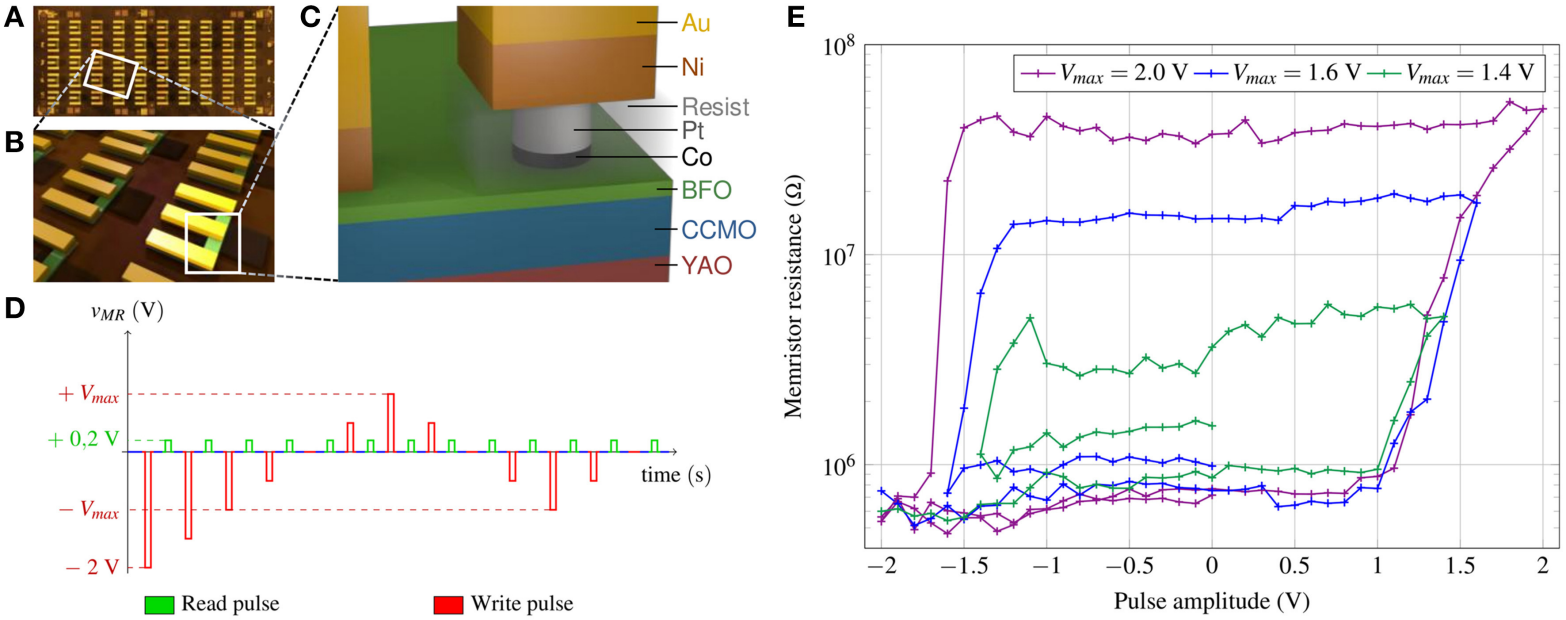
**(A)** Optical microscope image of the chip after patterning showing 5×10 ferroelectric tunnel junctions (FTJ); **(B)** 3D representation of a zoomed area containing a few FTJs. The three parallel bars are the ground-signal-ground contact pads; **(C)** 3D sketch of one FTJ (Boyn et al., 2014); **(D)** schema of the voltages applied to the memristor. The reading pulse V_read_ is lower than the threshold (V_read_ = 200 mV). Writing is performed by the application of 100 ns voltage pulses of different amplitudes. The writing voltages increase from −2 V to V_max_ by a step of 0.1 V. Then, the amplitude of the writing pulses decreases to −V_max_; **(E)** dependence of the resistance of the ferroelectric tunnel memristor measured at V_read_ on the applied writing cycles. The different curves correspond to different consecutive measurements with varying V_max_.

The “ferroelectric tunnel memristor” (Bibes et al., 2010) is based on an emerging digital memory concept, subject of intense academic and industrial developments, the ferroelectric resistive RAM (International Technology Roadmap For Semiconductors, 2011). Its base is the ferroelectric tunnel junction (FTJ): an insulating ultrathin (several nanometers) ferroelectric barrier sandwiched between two metallic electrodes (Figure 6A). Strain from the substrate assures that the ferroelectric polarization points to one of the electrodes. The polarization can be switched upon application of short voltage pulses and results in resistance changes of up to several orders of magnitude (Garcia et al., 2009; Chanthbouala et al., 2012a). This resistance contrast is linked to different polarization screening in the electrodes: the effective tunneling barrier height dependents on the direction of the ferroelectric layer's polarization and therefore strongly influences the tunneling current. Additionally, the strong non-linearity of the ferroelectric tunnel junction allows for a non-destructive resistance reading at low DC voltage.

By designing the devices in such way that the switching occurs through non-uniform ferroelectric domain configurations, quasi-analog resistance variations can be obtained (Chanthbouala et al., 2012b). A direct link between these intermediate resistance states and the ferroelectric domain configuration allows the description of its dynamic behavior through models of domain nucleation and growth in ferroelectric films. Furthermore, the cumulative behavior upon application of trains of voltage pulses has already been demonstrated. As the polarization reversal process in the ferroelectric film depends on pulse amplitude and duration, these parameters can be adapted to achieve the desired resistance change in the memristive device—a very promising feature for the implementation of STDP-based learning with ferroelectric tunnel memristors (Chanthbouala et al., 2012b).

It has recently been demonstrated that fully-patterned solid-state ferroelectric tunnel memristors based on BiFeO_3_ (fully patterned submicron Co/BiFeO_3_/Ca_0.96_Ce_0.04_MnO_3_ tunnel junctions) can be produced with high yield and with low device-to-device variations. They show resistance contrasts of more than 3 orders of magnitude, can be commuted with pulses of 100 ns and amplitudes of about 2 V, and have a large endurance of over 4 × 10^6^ cycles (Boyn et al., 2014).

In Figure 6, we plot as in Yamada et al. (2013) the multilevel behavior of a ferroelectric tunnel memristor depending on applied voltages. The curves in Figure 6B show the DC resistance value of the device after writing pulses of different amplitudes. To use this memristor as a plastic synapse we consider −*V*_*MR*_ to represent the time difference Δ*T* = *t*_*post*_ − *t*_*pre*_. Then Δ*T* > 0, i.e., *V*_*MR*_ < 0 in Figure 6B, implies increasing conductance that corresponds to Hebb's rule. Conversely, Δ*T* < 0 results in a decrease of the synaptic weight.

Choosing the waveform of Figure 3B1 for pre- and post-synaptic voltage neurons, the width of the positive square pulse can be as low as 100 ns in the case of the ferroelectric tunnel memristor. Accordingly, the ramp phase of the waveform will be a few times larger than this. As a result, the time difference between spikes for the STDP shown in Figure 3B2 can be less than 1 μs.

### Spin-transfer torque magnetic tunnel junction as a stochastic synapse

Spin-Transfer Torque Magnetic Tunnel Junctions (STT-MTJs) constitute another choice to implement plastic non-volatile synapses. They rely on a different operating mechanism than the devices presented in the rest of the paper, and for this reason are not always thought as memristive devices. Their specific stochastic behavior, however, can be particularly interesting for synaptic applications. And as they constitute the basic cell of the second generation of Spin Transfer Torque Magnetic RAM (STT-MRAM)—which is currently reaching the market—, they present a high level of CMOS compatibility and of maturity.

**Figure 7:**
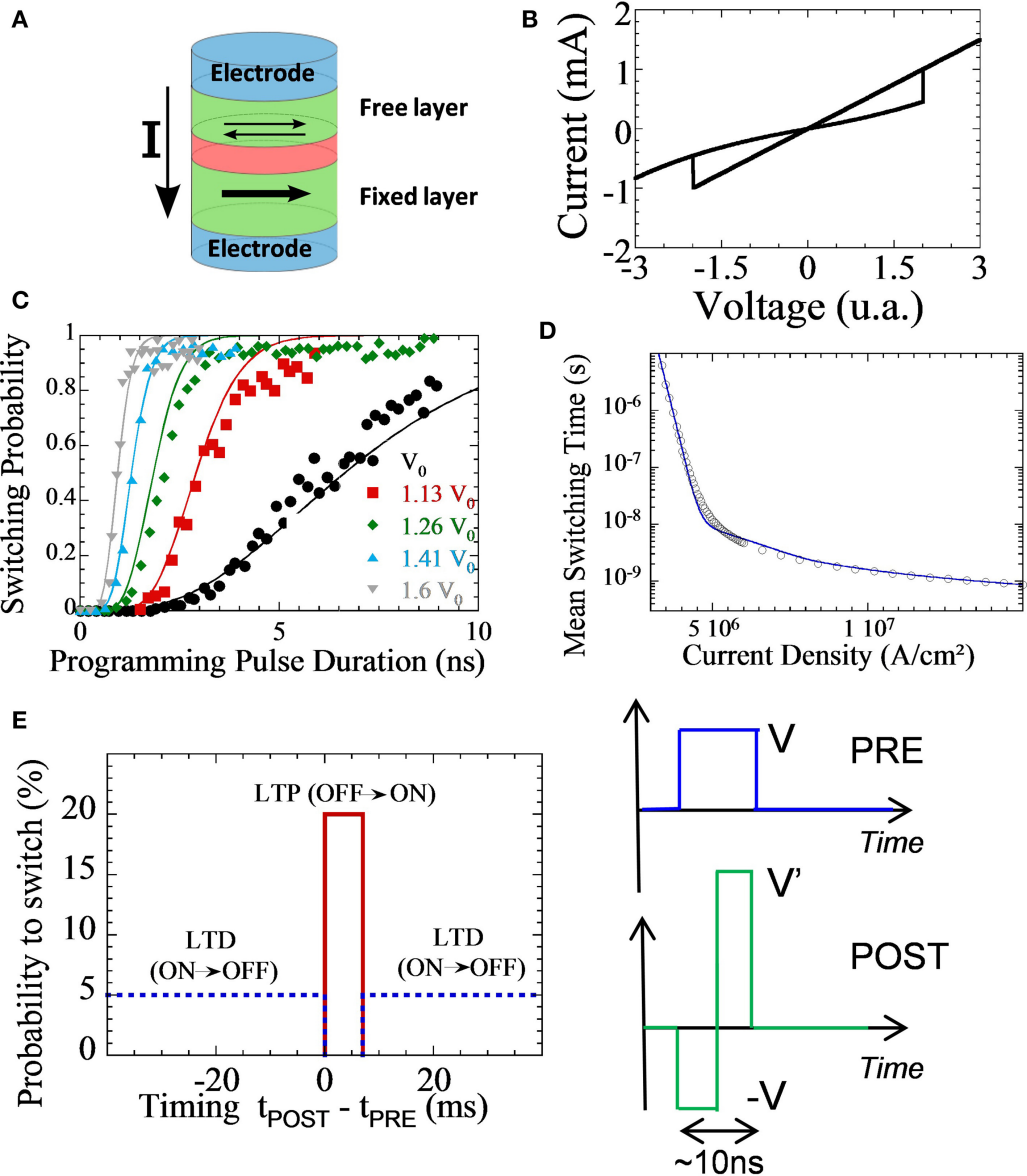
**From Vincent et al. (2014, 2015). (A)** Cartoon of a Spin Transfer Torque Magnetic Tunnel Junction (STT-MTJ). **(B)** Typical I–V curve of the STT-MTJ. **(C)** Experimental measurements of stochastic switching. **(D)** Model of mean switching time as a function of programming current. **(E)** Our simplified STDP rule and PRE and POST overlapping pulses which implement it naturally with STT-MTJs.

The basic structure of a STT-MTJ is presented in Figure 7A and is constituted by an ensemble of layers of different materials. The magnetic “fixed” layer is a small magnet whose magnetization is pinned in one direction. The magnetic “free” layer is a thinner magnet whose magnetization can be either parallel (P) or antiparallel (AP) to the one of the fixed layer. Due to the Tunnel Magnetoresistance effect, the electrical resistance of the P and AP state is different. And due to the Spin Transfer Torque effect, a positive current can switch the device from AP to P state, and a negative current can switch the device from P to AP state. This leads to the I–V curve seen in Figure 7B, which is reminiscent of a memristive device. However, MTJs are truly binary device: AP and P states are the only possible states. Some proposals exist to increase the number of states (Lou et al., 2008) or to include another physical effect (domain wall motion) in the MTJ to reach multilevel behavior (Wang et al., 2009; Chanthbouala et al., 2011). However, these variations do not exhibit the same degree of maturity as binary STT-MTJs. In comparison with traditional memristive devices, STT-MTJs are fast to write (programming can be as fast as 1.5 ns) and possess outstanding endurance (switching the free layer magnet is not associating with an aging mechanism). Their main drawback is a relatively high fabrication cost and a low R_OFF_/R_ON_ ratio. STT-MTJs should be associated with different CMOS circuits than other memristive devices for this reason (Zhang et al., 2014).

Additionally, a specificity of STT-MTJs, of special interest for synaptic applications, is that switching is stochastic. When one applies a programming pulse, a STT-MTJ has only a *probability* to switch state, which is independent of the STT-MTJ's history: every time the programming pulse is applied, the STT-MTJ has the same probability to switch state. This is well-seen on the experimental measurements of Figure 7C on devices of Devolder et al. (2008), Marins de Castro et al. (2012). The switching probability can be controlled by programming voltage and pulse duration. The basic physics behind this effect is well-understood (Diao et al., 2007; Devolder et al., 2008) and we have recently developed a comprehensive analytical model of it for circuits and systems designers (Vincent et al., 2015). As seen in Figure 7D, a striking feature is that the mean switching time of the STT-MTJ can be adjusted over many orders of magnitude by choosing the programming current. It has also been proven that STT-MTJ stochastic switching can be used to generate high quality random numbers that pass standardized statistical tests qualifying true random number generators (Fukushima et al., 2014). Stochastic switching can also be adjusted by layout of the junctions (size and eccentricity).

STT-MTJs are suitable for implementing a stochastic version of STDP that has been studied in several recent works (Kavehei, 2013; Suri et al., 2013; Yu et al., 2013; Vincent et al., 2014). They exploit, at the system level, a functional equivalence (Goldberg et al., 2001) that exists between multi-level deterministic synapses and binary probabilistic synapses. When a long term potentiation or depression occurs, instead of changing the conductance of the synapse partially, stochastic STDP has a small probability of changing it totally. And if several STT-MTJs are connected in parallel, a multibit synapse can be emulated. Since STT-MTJs have no internal dynamic besides stochastic switching, stochastic STDP can be implemented using similar strategies to the one used for ferroelectric devices. Only the behavior at the system level will be different.

In our works, we have been working with a stochastic version of the simplified version of STDP which is theorized in Nessler et al. (2013) and also used in Bichler et al. (2012a), Suri et al. (2013), Querlioz et al. (2013), and similar to the one of Figures 3G1,G2. A possible implementation with STT-MTJs is summarized on Figure 7E. It relies on overlapping pulses, but with clear separation of transmission and programming operation (Suri et al., 2013; Vincent et al., 2014). Although very simple, this STDP rule can lead to complex machine learning tasks like learning to detect cars on a video (Vincent et al., 2014). Additionally, we have observed that it is surprisingly robust to STT-MTJ variability (Vincent et al., 2014). However, this is just an example and other forms of STDP may be implemented with STT-MTJs if one accepts their stochastic nature.

## SRDP with memristive devices

The learning process described in the previous section has been implemented in a large variety of solid state memory devices with non-volatile characteristics. However, if we consider the synaptic plasticity mechanisms observed in biological computing systems, modification of the synaptic efficiency (evaluated by measuring the transmission of a single spike and equivalent to the synaptic weight) can be either permanent (i.e., lasting for months to years) or temporary (i.e., relaxing to its initial state with a characteristic time constant in the milliseconds to hours range). This observation leads to the definition of Long Term Plasticity (LTP) and Short Term Plasticity (STP), respectively. We can notice that the boundary classification into Long Term (LT) and Short Term (ST) effects is not well-defined and should be considered with respect to the required task. Both STP and LTP can correspond to an increase or decrease of the synaptic efficiency thus leading to the definition of Short Term (Long Term) potentiation and depression, respectively. In biology, synaptic plasticity can be attributed to various mechanisms involved in the transmission of the signal between a pre- and post-neuron, such as neurotransmitter release modification, neurotransmitter recovery in the pre-synaptic connection, receptors sensitivity modification or even structural modification of the synaptic connection (see Bliss and Collingridge, 1993), for a description of the different mechanisms involved in STP and LTP). Based on this observation, two important points need to be stressed. First, STP and LTP processes are not restricted to a particular learning strategy (i.e., STDP and SRDP, for example). In this section, we present examples of STP and LTP processes based on a particular case of rate coding strategy but these considerations are still valid for other coding strategies (see Alibart et al., 2012, for STDP with STP devices). Secondly, if plasticity is intimately linked to the notion of learning, it is important to notice that there is no one-to-one equivalence between the concepts of STP, LTP and the notion of Short Term Memory (STM) and Long Term Memory (LTM). Indeed, even if a direct parallel has been proposed based on the particular concept of memory consolidation (Lamprecht and Ledoux, 2004), which corresponds to accumulation of Short Term effect leading to Long Term memory, there are still very important questions to be answered about how learning (and the associated synaptic plasticity) is related to the memorization of information that can also present different time scale from milliseconds to years.

### Short term plasticity (STP)

**Figure 8:**
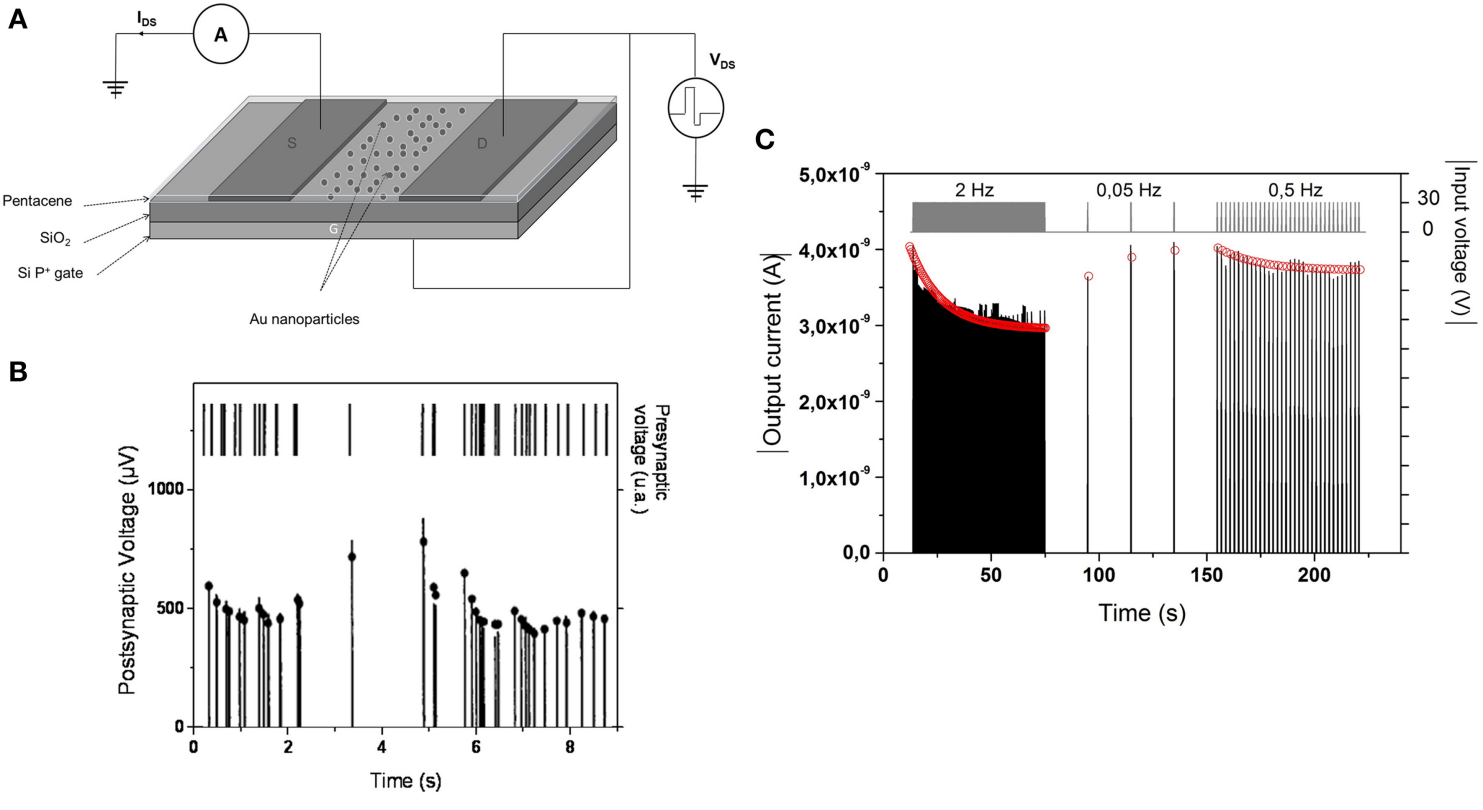
**(A)** Schematic of the NOMFET in a diode-like configuration. This leaky memory transistor was proposed to implement the Short Term Plasticity **(B)** STP measured in biological synapses (Varela et al., 1997) and **(C)** STP implement in solid state device.

Implementation of STP has been proposed in a variety of nanoscale memory devices. The first proposition of STP was realized in a nanoparticles/organic memory transistor (NOMFET)—Figure 8 (Alibart et al., 2010). The basic principle of this device is equivalent to a floating gate transistor. Charges are stored in the nanoparticles and modify the channel conductivity via Coulombic repulsion between the carriers (holes) and the charged nanoparticles. The particularity of this device is to present a leaky memory behavior: charges stored in the nanoparticles tend to relax with a characteristic time constant in the 1–100 s range. When the NOMFET is connected in a diode like configuration (Figure 8A), each input spike (with a negative voltage value) charges the nanoparticles and decreases the NOMFET conductivity. Between pulses, charges escape from the nanoparticles and the conductivity relaxes toward its resting value. By analogy with biology, this device mimics the STP observed in depressing synapses (Figures 8B,C) and described by Abbott et al. (1997). As a matter of comparison, this synaptic functionality is realized with a single memory transistor when its implementation in Si based technologies (i.e., CMOS) required 7 transistors (Boegerhausen et al., 2003). STP has been also demonstrated in two-terminal devices that would ensure higher device density when integrated into complex systems. Equivalently, STP in two terminals devices is implemented by taking advantage of the volatility of the different memory technologies (i.e., low retention of the state that is often a drawback in pure memory applications). Cationic redox systems based on Electro-Chemical Memory cells (ECM) (Ohno et al., 2011) or anionic Valence Change Memory (Chang et al., 2011; Yang et al., 2013) have demonstrated STP with a facilitating behavior. In such devices, Short Term Plasticity is ensured by the low stability of the conducting filaments that tend to dissolve, thus relaxing the device toward the insulating state. TiO2 VCM cells have been reported with both facilitating and depressing behavior (Lim et al., 2013) with relaxation related to oxidation-reduction counter reaction. Protonic devices have demonstrated STP with depressing functionality due to proton recovery latency from atmosphere required to restore the proton concentration and conductivity (Josberger et al., 2014).

In the case of rate dependent plasticity, STP can be of depressing type (i.e., decrease of the synaptic efficiency when synaptic activity increase) or facilitating type (i.e., increase of synaptic efficiency when synaptic activity increase). In terms of functionality, Abbott et al. (1997) has demonstrated that depressing synapses with STP act as a gain control device (at high frequency, i.e., high synaptic activity, the synaptic weight is decreased, thus leading to a reduction of the signal when activity becomes too important). More generally, STP (both depressing and facilitating) provides a very important frequency coding property (as depicted in Figures 8B,C) that could play a major role in the processing of spike-rate coded information. Indeed, if a simple Integrate and Fire neuron (I&F) is associated with static weight (with no dependence with spike frequency), the computing node (i.e., neuron and synapses) is only a linear filter (linear combination of the different input) while STP turns the node to non-linear. This property can be used to implement reservoir computing approaches as proposed by Maass (Buonomano and Maass, 2009) with the Liquid State Machine and could be an important property of biological systems for computation.

### Co-existence of STP and LTP in the same device

**Figure 9:**
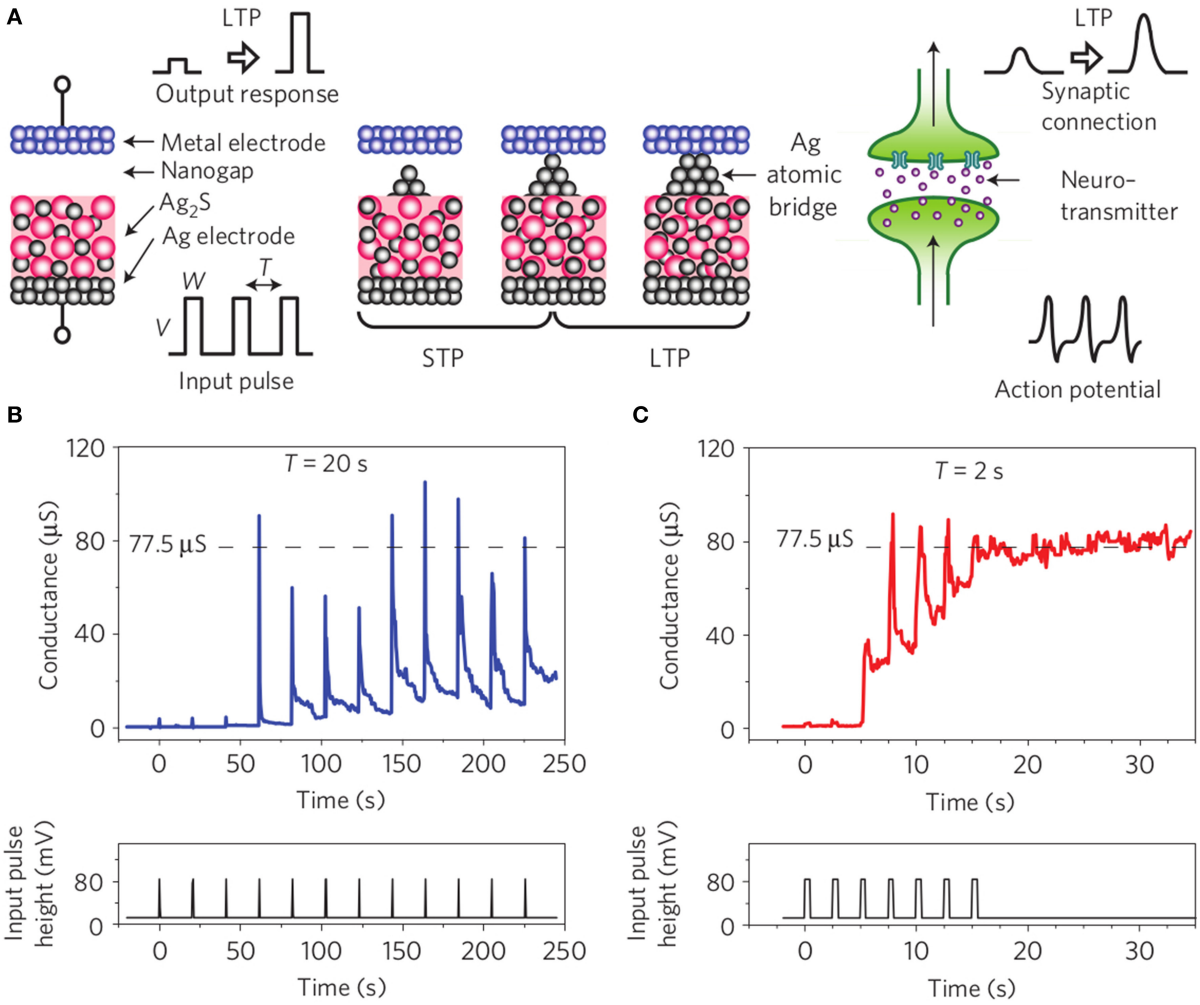
**Adapted from Ohno et al. (2011). (A)** Schematic of atomic bridge devices that was proposed for Short Term Plasticity, Long Term Plasticty (STP/LTP) transition demonstration. Depending on the spiking activity, **(B)** the metallic filament do not bridge the two electrodes and tends to relax toward the OFF state while it remains **(C)** in the ON state once it bridges the two electrodes.

If the contribution of ST and LT processes to computing is not completely understood in biological systems, we should consider that both STP and LTP effects in synaptic connections are required in neuro-inspired computing systems. A first approach is to consider that repetition of short term effects should lead to Long Term modification in the synaptic connections. This behavior would explain the important hypothesis of memory consolidation in the sense of psychology (Lamprecht and Ledoux, 2004). Ohno et al. (2011) reported the coexistence of Long Term and Short Term Potentiation in atomic bridge technology (Figure 9). Depending on pre-synaptic activity (associated to spike rate in this case), the synaptic conductivity is increased due to the formation of a Ag filament across the insulating gap. While for low frequency, the bridge tends to relax between pulses, higher frequencies lead to a strong filament that maintains the device in the ON state. These results suggest a critical size of the bridging filament in order to maintain the conductive state (i.e., providing a LTP of the synaptic connection). Similar results have been obtained in a variety of memory devices where filamentary switching displayed two regimes of volatility. Chang et al. (2011) have evidenced a continuous evolution of the volatility as a function of the conductivity level of the device in WO3 oxide cells attributed to the competition between oxygen vacancies drift (creation of conductive path across the device) and lateral diffusion (disruption of the conducting filaments). Another description of these two regimes of volatility could be associated to a competition between surface and volume energies in the conductive filament.

If this transition between Short Term Plasticity and Long Term Plasticity is intuitively well-associated to the concept of STM to LTM learning in psychology, we can note that it induces some restriction in term of network functionality. Indeed, in biology, the facilitating process observed at short time scale and associated to an increase of neurotransmitter release probability during a burst of spike (i.e., corresponding to an increase of synaptic efficiency at high frequency spiking rate) is additive with LTP (Bliss and Collingridge, 1993). In this case the node (neuron and synapses) maintains its rate coding property (associated to short term process and described previously as a non-linear node) and can also display long term modification of the synaptic weight. Alternative approaches are still needed as proposed by Cantley et al. (2011) where Short Term processes and Long Term Processes are realized by two different devices (leaky floating gate transistor and non-volatile two-terminal devices) in order to match the complexity of biological synapses. One fundamental issue that needs to be explored is the balance between the device functionality required for proper operation of computing systems (i.e., performances) and optimal integration in order to match synaptic density required for computing.

## Toward memristor-CMOS architectures and circuits

In order to exploit the plasticity of memristor-based artificial synapses, specific circuit architecture needs to be developed. Indeed, depending on the polarity and electrical characteristics of investigated devices, two types of circuits have been identified which are described in the following paragraphs.

### Circuits for bipolar memristors

Most of the works on memristive devices that have been published over the last couple of years focus on bipolar resistive switching devices (Waser and Aono, 2007; Snider, 2008; Strukov et al., 2008; Jo et al., 2010). This is the case for all the devices presented in Section STDP Learning Thanks to Overlapping Events. These devices exhibit characteristics close to the original Memristor predicted by Chua. Their resistance can be increased or decreased with opposite polarity voltage pulses and the resistance change is cumulative with the previous state of the device, which makes them particularly suitable to implement synaptic-like functionality.

**Figure 10:**
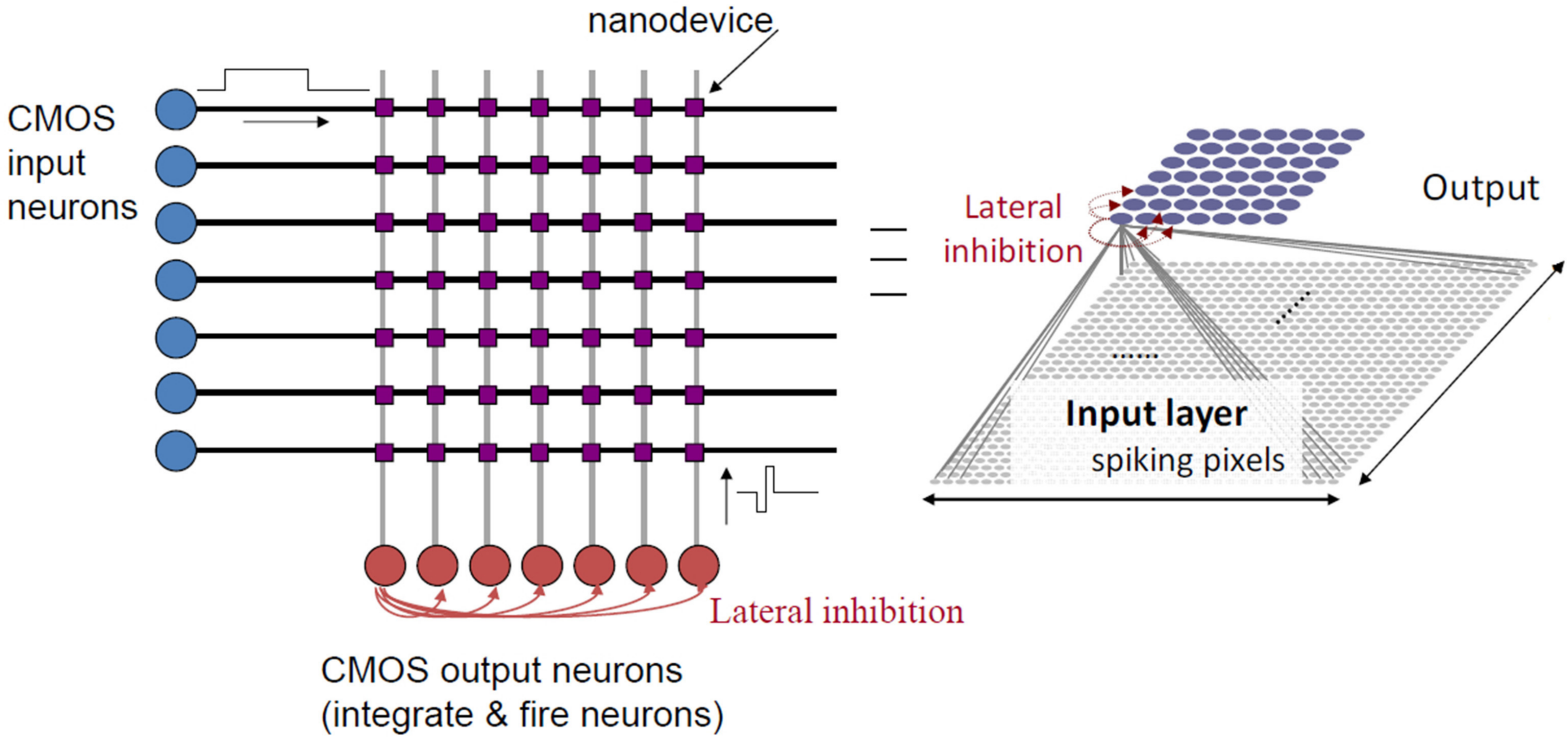
**Basic crossbar circuit topology.** Wires originate from CMOS input layer (horizontal black wires) and from the CMOS output layer (vertical gray wires). Memristive nanodevices are located at the cross points of the horizontal and vertical wires.

A biologically-inspired spiking NN-based computing paradigm which exploits the specific physics of those devices is presented in Querlioz et al. (2011, 2013). In this approach, CMOS input and output neurons are connected by bipolar memristive devices used as synapses. It is natural to lay out the nanodevices in the widely studied crossbar as illustrated on Figure 10. Learning is competitive thanks to lateral inhibition and fully unsupervised using a simplified form of STDP.

Using this topology, performance comparable to traditional supervised networks has been measured (Querlioz et al., 2013) for the textbook case of character recognition, despite extreme variations of various memristive device parameters. With the same approach, unsupervised learning of temporally correlated patterns from a spiking silicon retina has also been demonstrated. When tested with real-life data, the system is able to extract complex and overlapping temporally correlated features such as car trajectories on a freeway (Bichler et al., 2012a).

### Circuits for unipolar memristors

All that we have discussed in this work can be adapted to another class of memristive devices—the unipolar devices where all applied voltages to increase or decrease the resistance value are positive. Among them, in particular, Phase-Change Memory (PCM) has good maturity, scaling capability, high endurance, and good reliability (Fantini et al., 2010). PCM resistance can be modified by applying a temporal temperature gradient modifying the material organization between an amorphous and a crystalline phase. The amorphous region inside the phase change layer can be crystallized by applying set pulses, thus increasing device conductance. It was shown that the magnitude of the relative increase in conductance can be controlled by the pulse amplitude and by the equivalent pulse width (Kuzum et al., 2012). Amorphization, on the other hand, is a more power-hungry process and is not progressive with identical pulses. The current required for amorphization is typically 5–10 times higher than for crystallization, even for state-of-the art devices.

**Figure 11:**
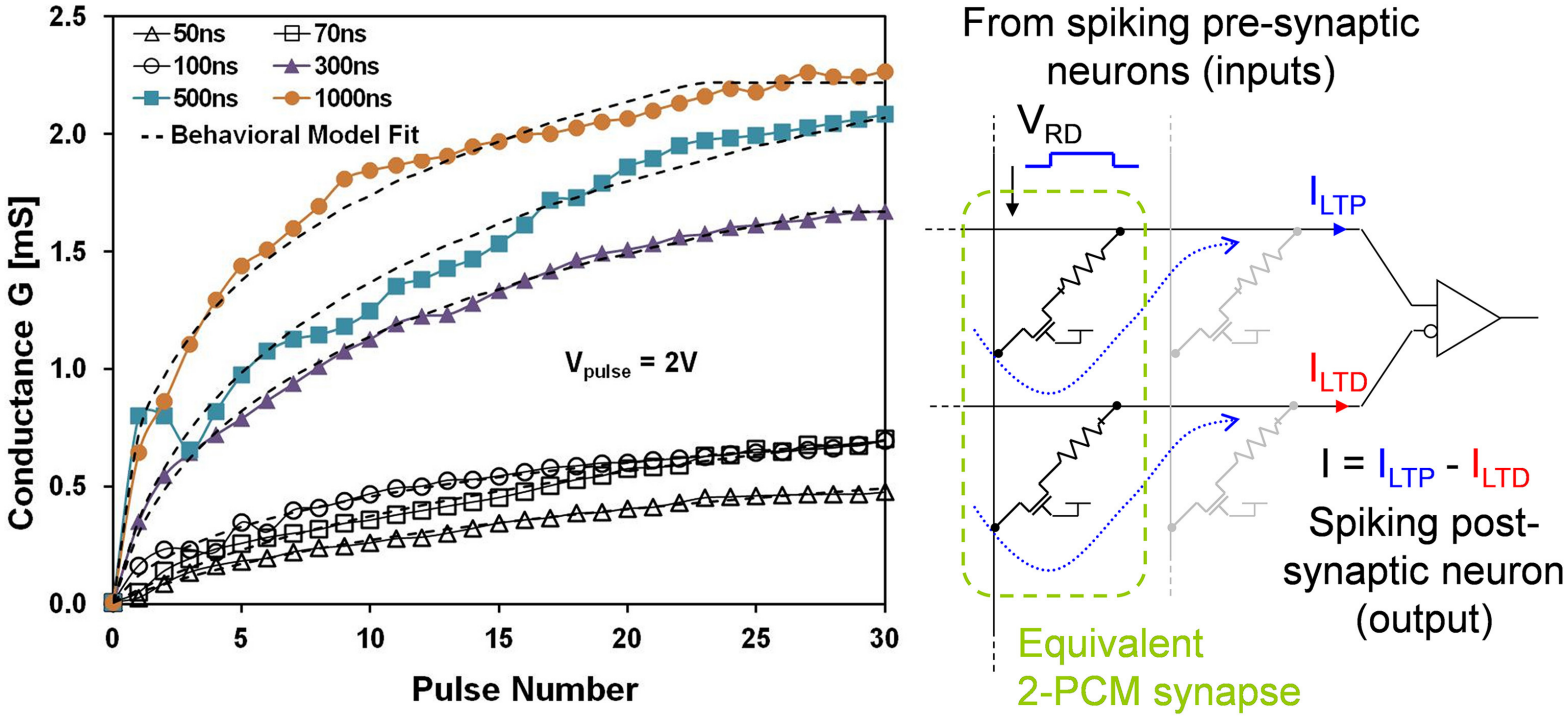
**Left:** Experimental LTP characteristics of the unipolar PCM device. **Right:** Principle of an equivalent bipolar synapse realized with a 2-PCM circuit. Note that the neuron circuit is not represented on the schematic.

To overcome these issues, a novel low-power architecture “2-PCM Synapse” was introduced in Bichler et al. (2012b). The idea is to emulate synaptic functions in large scale neural networks using two PCM devices constituting one synapse as shown in Figure 11. These two devices have an opposite contribution to the neuron's integration. When the synapse needs to be potentiated, the Long Term Potentiation (LTP) PCM device undergoes a partial crystallization, increasing the equivalent weight of the synapse. Similarly, when the synapse must be depressed, the Long Term Depression (LTD) PCM device is crystallized. As the LTD device has a negative contribution to the neuron's integration, the equivalent weight of the synapse is reduced. Furthermore, because gradual crystallization is achieved with successive identical voltage pulses, the pulse generation is greatly simplified. Note however that such synaptic circuit will require a slightly more complex post-synaptic neuron circuit in order to deal with pulse integration and generation. This should have a limited impact on the overall neuromorphic circuit given the lower number of neurons vs. synapses.

## Discussion

Memristive devices are an appealing solution to implement plastic synapses, if we develop the specific driving signals to emulate different learning rules. The most popular synaptic plasticity implementation is based on the realization of Hebbian learning, and in particular of STDP. We shall however note that other plasticity mechanisms exist that have been studied and modeled as suggested in a recent work (Kornijcuk et al., 2014). In this paper, we focused on different implementations of STDP, by taking advantage of the device physics of different memristive devices. The functional differences in the behaviors of the devices directly translate into differences in the learning rules (real time or accelerated, deterministic or stochastic). Using other devices, we also presented other synaptic ideas, such as short term plasticity, or those which exploit interactions between short term and long term plasticity. Finally, we proposed some implementation ideas, offering a large overview of the different possibilities in several material systems.

As memristors are primarily targeted toward future high-density nanoscale arrays, CMOS driver circuits need to be scaled to these dimensions as well. That is to say, the required neuromorphic driver circuits need to be moved to deep submicron technologies. One recently presented method to achieve this is the use of switched-capacitor neuromorphic circuits, which are able to implement the required analog waveforms in high density technologies as small as 28 nm (Mayr et al., 2014b). Coupled with deep submicron CMOS sensors (Henker et al., 2007), they offer the possibility of a full image processing pyramid based on memristive computation in a nanoscale CMOS-memristor hybrid. However, developing appropriate and highly scaled driver circuits for memristive synapses which do not bring large overheads is a significant goal for today's research. This is especially true for proposals that exploit passive crossbar integration. Such circuit topology is particularly appealing for neuromorphic engineers as it offers a direct equivalent for the neuron/synapse circuit with high parallelism and high integration density in which a single device is associated to a single synapse between two neurons (input line and output column). However, it brings circuit challenges (crosstalk, sneak path, impedance mismatch,…) that need to be overcome.

From a more systems' perspective, the most interesting applications for nanoscale memristors will be those that require a large number of learned or programmed synaptic weights. It is important to already consider such applications, to understand the true impact of memristive technology. One of these applications is the Neural Engineering Framework (Eliasmith and Anderson, 2004), which can be used to implement straightforward signal computation, sensor fusion (Mayr et al., 2014a), and recognition (Bichler et al., 2012a), but also models of cognition (Eliasmith et al., 2012). The large number of synapses offered by nanoscale memristive arrays makes the implementation of complex cognitive processing of such large-scale models (Eliasmith et al., 2012) on a single CMOS-memristor hybrid IC a real possibility.

Finally, it is important to understand that there are no absolute optimal memristive devices for the implementation of plasticity in hardware neural networks. The variety of behaviors observed in today's research will be an advantage for neuromorphic chip designers and computational neuroscientists since it opens new paths of implementation of neural computations. In this respect, the plastic behaviors measured on memristive devices and presented in this paper provide the primitive for future neuromorphic breakthroughs.

### Conflict of interest statement

The authors declare that the research was conducted in the absence of any commercial or financial relationships that could be construed as a potential conflict of interest.

## References

[B1] Abbott L. F. Varela J. A. Sen K. Nelson S. B. (1997). Synaptic depression and cortical gain control. Science 275, 221–224. 10.1126/science.275.5297.221 8985017

[B2] Alibart F. Pleutin S. Bichler O. Gamrat C. Serrano-Gotarredona T. Linares-Barranco B. (2012). A memristive nanoparticle/organic hybrid synapstor for neuroinspired computing. Adv. Funct. Mater. 22, 609–616 10.1002/adfm.201101935

[B3] Alibart F. Pleutin S. Guérin D. Novembre C. Lenfant S. Lmimouni K. (2010). An organic nanoparticle transistor behaving as a biological spiking synapse. Adv. Funct. Mater. 19, 330–337 10.1002/adfm.200901335

[B4] Baek I. G. Lee M. S. Seo S. Lee M. J. Seo D. H. Suh D.-S. (2004). Highly scalable nonvolatile resistive memory using simple binary oxide driven by asymmetric unipolar voltage pulses. Tech. Dig. IEEE Int. Electron Devices Meet. 587–590 10.1109/IEDM.2004.1419228

[B5] Bi G. Poo M. (1998). Synaptic modifications in cultured hippocampal neurons: dependence on spike timing, synaptic strength, and postsynaptic cell type. J. Neurosci. 18, 10464–10472. 9852584 10.1523/JNEUROSCI.18-24-10464.1998 PMC6793365

[B6] Bi G. Poo M. (2001). Synaptic modification by correlated activity: Hebb's postulate revisited. Annu. Rev. Neurosci. 24, 139–166. 10.1146/annurev.neuro.24.1.139 11283308

[B7] Bibes M. Grollier J. Barthélémy A. Mage J.-C. (2010). Ferroelectric Device with Adjustable Resistance. Patent WO 2010142762 A1.

[B8] Bichler O. Querlioz D. Thorpe S. J. Bourgoin J.-P. Gamrat C. (2012a). Extraction of temporally correlated features from dynamic vision sensors with spike-timing-dependent plasticity. Neural Netw. 32, 339–348. 10.1016/j.neunet.2012.02.022 22386501

[B9] Bichler O. Suri M. Querlioz D. Vuillaume D. DeSalvo B. Gamrat C. (2012b). Visual pattern extraction using energy-efficient “2-PCM synapse” neuromorphic architecture. IEEE Trans. Electron Devices 59, 2206–2214 10.1109/TED.2012.2197951

[B10] Bliss T. V. P. Collingridge G. L. (1993). A synaptic model of memory: long-term potentiation in the hippocampus. Nature 361, 31. 10.1038/361031a0 8421494

[B11] Boegerhausen M. Suter P. Liu S.-C. (2003). Modeling short-term synaptic depression in silicon. Neural Comput. 15, 331–348. 10.1162/089976603762552942 12590810

[B12] Boyn S. Girod S. Garcia V. Fusil S. Xavier S. Deranlot C. (2014). High-performance ferroelectric memory based on fully patterned tunnel junctions. Appl. Phys. Lett. 104, 052909 10.1063/1.4864100

[B13] Buonomano D. V. Maass W. (2009). State-dependent computations: spatiotemporal processing in cortical networks. Nat. Rev. Neurosci. 10, 113–125. 10.1038/nrn2558 19145235

[B14] Cantley K. D. Subramaniam A. Stiegler H. J. Chapman R. A. Vogel E. M. (2011). Hebbian learning in spiking neural networks with nanocrystalline silicon TFTs and memristive synapses. IEEE Trans. Nanotechnol. 5, 1066 10.1109/TNANO.2011.2105887 24808583

[B15] Cassenaer S. Laurent G. (2007). Hebbian STDP in mushroom bodies facilitates the synchronous flow of olfactory information in locusts. Nature 448, 709–713. 10.1038/nature05973 17581587

[B16] Cederstroem L. Starke P. Mayr C. Shuai Y. Schmidt H. Schüffny R. (2013). A model based comparison of BiFeO3 device applicability in neuromorphic hardware. IEEE Int. Symp. Circuits Syst. 2323–2326 10.1109/ISCAS.2013.6572343

[B17] Chang T. Jo S.-H. Lu W. (2011). Short term memory to long term memory transition in a nanoscale memristor. ACS Nano. 5, 7669–7676. 10.1021/nn202983n 21861506

[B18] Chanthbouala A. Crassous A. Garcia V. Bouzehouane K. Fusil S. Moya X.. (2012a). Solid-state memories based on ferroelectric tunnel junctions. Nature Nano. 7, 101. 10.1038/nnano.2011.213 22138863

[B19] Chanthbouala A. Garcia V. Cherifi R. O. Bouzehouane K. Fusil S. Moya X.. (2012b). A ferroelectric memristor. Nature Mater. 11, 860–864. 10.1038/nmat3415 22983431

[B20] Chanthbouala A. Matsumoto R. Grollier J. Cros V. Anane A. Fert A. (2011). Vertical-current-induced domain-wall motion in MgO-based magnetic tunnel junctions with low current densities. Nat. Phys. 7, 626–630 10.1038/nphys1968

[B21] Chua L. (1971). Memristor-missing circuit element. IEEE Trans. Circuit Theor. 18, 507–519. 10.1109/TCT.1971.108333718451858

[B22] Chua L. (2014). If it's pinched it's a memristor. Semicond. Sci. Technol. 29:104001 10.1088/0268-1242/29/10/104001

[B23] Chua L. O. Kang S. M. (1976). Memristive devices and systems. Proc. IEEE 64, 209–223 10.1109/PROC.1976.10092

[B24] Devolder T. Hayakawa J. Ito K. Takahashi H. Ikeda S. Crozat P.. (2008). Single-shot time-resolved measurements of nanosecond-scale spin-transfer induced switching: stochastic versus deterministic aspects. Phys. Rev. Lett. 100:057206. 10.1103/PhysRevLett.100.057206 18352422

[B25] Diao Z. Li Z. Wang S. Ding Y. Panchula A. Chen E. (2007). Spin-transfer torque switching in magnetic tunnel junctions and spin-transfer torque random access memory. J. Phys. Condens. Matter. 19:165209 10.1088/0953-8984/19/16/165209

[B26] Eisenreich H. Mayr C. Henker S. Wickert M. Schüffny R. (2009). A novel ADPLL design using successive approximation frequency control. Microelectron. J. 40, 1613–1622 10.1016/j.mejo.2008.12.005

[B27] Eliasmith C. Anderson C. C. H. (2004). Neural Engineering: Computation, Representation, and Dynamics. Neurobiological Systems. Cambridge, MA: MIT Press.

[B28] Eliasmith C. Stewart T. C. Choo X. Bekolay T. DeWolf T. Tang Y.. (2012). A large-scale model of the functioning brain. Science 338, 1202–1205. 10.1126/science.1225266 23197532

[B29] Fantini A. Sousa V. Perniola L. Gourvest E. Bastien J. C. Maitrejean S. (2010). N-doped GeTe as performance booster for embedded phase-change memories, in International Electron Devices Meeting (San Francisco, CA), 29.1.1–29.1.4 10.1109/IEDM.2010.5703441

[B30] Feldman D. (2000). Timing-based LTP and LTD at vertical inputs to layer II/III pyramidal cells in rat barrel cortex. Neuron 27, 45–56. 10.1016/S0896-6273(00)00008-8 10939330

[B31] Fieres J. Schemmel J. Meier K. (2008). Realizing biological spiking network models in a configurable wafer-scale hardware system, in IEEE International Joint Conference Neural Network (Hong Kong), 969–976 10.1109/IJCNN.2008.4633916

[B32] Finelli L. A. Haney S. Bazhenov M. Stopfer M. Sejnowski T. J. (2008). Synaptic learning rules and sparse coding in a model sensory system. PLoS Comput. Biol. 4:e1000062. 10.1371/journal.pcbi.1000062 18421373 PMC2278376

[B33] Flocke A. Noll T. G. (2007). Fundamental analysis of resistive nanocrossbars for the use in hybrid nano/cmos-memory, Proceedings of 33rd ESSCIRC. 328–331 10.1109/ESSCIRC.2007.4430310

[B35] Froemke R. Dan Y. (2002). Spike-timing-dependent synaptic modification induced by natural spike trains. Nature 416, 433–438. 10.1038/416433a 11919633

[B36] Fukushima A. Seki T. Yakushiji K. Kubota H. Imamura H. Yuasa S. (2014). Spin dice: a scalable truly random number generator based on spintronics. Appl. Phys. Express. 7:083001 10.7567/APEX.7.083001

[B37] Garcia V. Fusil S. Bouzehouane K. Enouz-Vedrenne S. Mathur N. D. Barthélémy A.. (2009). Giant tunnel electroresistance for non-destructive readout of ferroelectric states. Nature 460, 81–84. 10.1038/nature08128 19483675

[B38] Gerstner W. Kempter R. Leo van Hemmen J. Wagner H. (1996). A neuronal learning rule for sub-millisecond temporal coding. Lett. Nat. 383, 76–78. 10.1038/383076a0 8779718

[B39] Gerstner W. Ritz R. Hemmen J. L. (1993). Why spikes? Hebbian learning and retrieval of time-resolved excitation patterns. Biol. Cybern. 69, 503–515. 10.1007/BF00199450 7903867

[B40] Goldberg D. H. Cauwenberghs G. Andreou A. G. (2001). Probabilistic synaptic weighting in a reconfigurable network of VLSI integrate-and-fire neurons. Neural Netw. 14, 781–793. 10.1016/S0893-6080(01)00057-0 11665770

[B41] Hebb D. O. (1949). The Organization of Behavior. New York: Wiley and Sons.

[B42] Henker S. Mayr C. Schlüssler J.-U. Schüffny R. Ramacher U. Heittmann A. (2007). Active pixel sensor arrays in 90/65nm CMOS-technologies with vertically stacked photodiodes, in Proceedings if IEEE International Image Sensor Workshop. 16–19.

[B43] Indiveri G. Chicca E. Douglas R. (2006). A VLSI array of low-power spiking neurons and bistable synapses with spike-timing dependent plasticity. IEEE Trans. Neural Netw. 17, 211–221. 10.1109/TNN.2005.860850 16526488

[B34] International Technology Roadmap For Semiconductors (ITRS). (2011). Emerging Research Devices.

[B44] Jacob V. Brasier D. J. Erchova I. Feldman D. Shulz D. E. (2007). Spike-timing-dependent synaptic depression in the in vivo barrel cortex of the rat. J. Neurosci. 27, 1271–1284. 10.1523/JNEUROSCI.4264-06.2007 17287502 PMC3070399

[B45] Jeong D. S. Kim I. Ziegler M. Kohlstedt H. (2013). Towards artificial neurons and synapses: a materials point of view. RSC Adv. 3, 3169 10.1039/c2ra22507g

[B46] Jo S. H. Chang T. Ebong I. Bhadviya B. B. Mazumder P. Lu W. (2010). Nanoscale memristor device as synapse in neuromorphic systems. Nano Lett. 10, 1297–1301. 10.1021/nl904092h 20192230

[B47] Josberger E. E. Deng Y. Sun W. Kautz R. Rolandi M. (2014). Two terminal protonic devices with synaptic like short term depression and device memory. Adv. Mater. 26, 4986–4990. 10.1002/adma.201400320 24789251

[B48] Joubert A. Belhadj B. Temam O. Heliot R. (2012). Hardware spiking neurons design: analog or digital? in International Joint Conference Neural Network (Brisbane, QLD), 1–5 10.1109/IJCNN.2012.6252600

[B49] Kavehei O. (2013). Highly scalable neuromorphic hardware with 1-bit stochastic nano-synapses. IEEE Int. Symp. Circuits Syst. 1648–1651 10.1109/ISCAS.2014.6865468

[B50] Kawahara A. Azuma R. Ikeda Y. Kawai K. Katoh Y. Tanabe K. (2012). An 8Mb multi-layered cross-point ReRAM macro with 443MB/s write throughput, in IEEE International Solid-State Circuits Conference 432–434 10.1109/ISSCC.2012.6177078

[B51] Khan M. Lester D. Plana L. Rast A. Jin X. Painkras E. (2008). Spinnaker: mapping neural networks onto a massively-parallel chip multiprocessor, in IEEE International Joint Conference Neural Network (Hong Kong), 2849–2856 10.1109/IJCNN.2008.4634199

[B52] Kornijcuk V. Kavehei O. Lim H. Seok J. Y. Kim S. K. Kim I.. (2014). Multiprotocol-induced plasticity in artificial synapses. Nanoscale 6, 15151–15160. 10.1039/c4nr03405h 25373422

[B53] Kuzum D. Jeyasingh R. G. D. Lee B. Wong H.-S. P. (2012). Nanoelectronic programmable synapses based on phase change materials for brain-inspired computing. Nanoletters 12, 2179–2186. 10.1021/nl201040y 21668029

[B54] Kuzum D. Yu S. Wong H. P. (2013). Synaptic electronics: materials, devices and applications. Nanotechnology 24:382001. 10.1088/0957-4484/24/38/382001 23999572

[B55] Lamprecht R. Ledoux J. (2004). Structural plasticity and memory. Nat. Rev. Neurosci. 5, 45–54. 10.1038/nrn1301 14708003

[B56] Lau C. N. Stewart D. R. Williams R. S. Bockrath M. (2004). Direct observation of nanoscale switching centers in metal/molecule/metal structures. Nano Lett. 4, 569–572 10.1021/nl035117a

[B57] Lee H. Y. Chen P. S. Wu T. Y. Chen Y. S. Wang C. C. Tzeng P. J. (2008). Low power and high speed bipolar switching with a thin reactive Ti buffer layer in robust HfO2 based RRAM, in Technology Digital IEEE International Electron Devices Meeting (San Francisco, CA), 1–4 10.1109/IEDM.2008.4796677

[B58] Lim H. Kim I. Kim J.-S. Hwang C. S. Jeong D. S. (2013). Short term memory of TiO2 based electrochemical capacitors: empirical analysis with adoption of a sliding threshold. Nanotechnology 24:384005. 10.1088/0957-4484/24/38/384005 23999153

[B59] Linares Barranco B. Serrano-Gotarredona T. (2009b). Exploiting memristance in adaptive asynchronous spiking neuromorphic nanotechnology systems, 9th IEEE Conference Nanotechnology (Genoa), 601–604.

[B60] Linares-Barranco B. Serrano-Gotarredona T. (2009a). Memristance can explain spike-time-dependent-plasticity in neural synapses. Nat. Proc. NPRE.

[B61] Linn E. Rosezin R. Kügeler C. Waser R. (2010). Complementary resistive switches for passive nanocrossbar memories. Nat. Mater. 9, 403–406. 10.1038/nmat2748 20400954

[B62] Liu T.-Y. Yan T. H. Scheuerlein R. Chen Y. Lee J. K. Y. Balakrishnan G. (2013). A 130.7 mm^2^ 2-layer 32Gb ReRAM memory device in 24nm technology. IEEE Int. Solid-State Circuits Conf. 432–434, 210–212. 10.1109/ISSCC.2013.6487703

[B63] Livi P. Indiveri G. (2009). A current-mode conductance-based silicon neuron for address-event neuromorphic systems. Int. Symp. Circuits Syst. 2898–2901 10.1109/ISCAS.2009.5118408

[B64] Lou X. Gao Z. Dimitrov D. V. Tang M. X. (2008). Demonstration of multilevel cell spin transfer switching in MgO magnetic tunnel junctions. Appl. Phys. Lett. 93, 242502 10.1063/1.3049617

[B65] Marins de Castro M. Sousa R. C. Bandiera S. Ducruet C. Chavent A. Auffret S. (2012). Precessional spin-transfer switching in a magnetic tunnel junction with a synthetic antiferromagnetic perpendicular polarizer. J. Appl. Phys. 111, 07C912–07C912-3 10.1063/1.3676610

[B66] Markram H. Lübke J. Frotscher M. Sakmann B. (1997). Regulation of synaptic efficacy by coincidence of postsynaptic APS and EPSPS. Science 275, 213–215. 10.1126/science.275.5297.213 8985014

[B67] Masquelier T. Guyonneau R. Thorpe S. J. (2008). Spike timing dependent plasticity finds the start of repeating patterns in continuous spike trains. PLoS ONE 3:e1377. 10.1371/journal.pone.0001377 18167538 PMC2147052

[B68] Masquelier T. Guyonneau R. Thorpe S. J. (2009). Competitive STDP-based spike pattern learning. Neural Comp. 21, 1259–1276. 10.1162/neco.2008.06-08-804 19718815

[B69] Mayr C. Ehrlich M. Henker S. Wendt K. Schüffny R. (2007). Mapping complex, large scale spiking networks on neural VLSI. Int. J. Appl. Sci. Eng. Technol. 4, 37–42.

[B70] Mayr C. Noack M. Partzsch J. Schüffny R. (2010). Replicating experimental spike and rate based neural learning in CMOS. IEEE Int. Symp. Circuits Systems. 105–108 10.1109/ISCAS.2010.5537009

[B71] Mayr C. Partzsch J. (2010). Rate and pulse based plasticity governed by local synaptic state variables. Front. Synaptic Neurosci. 2:33. 10.3389/fnsyn.2010.00033 21423519 PMC3059700

[B72] Mayr C. Partzsch J. Noack M. Hänzsche S. Scholze S. Höppner S.. (2014b). A biological real time neuromorphic system in 28nm CMOS using low leakage switched capacitor circuits. Trans. Biomed. Circuits Syst. 10.1109/TBCAS.2014.2379294 25680215

[B73] Mayr C. Partzsch J. Noack M. Schüffny R. (2014a). Configurable analog-digital conversion using the neural engineering framework. Front. Neurosci. 8:201. 10.3389/fnins.2014.00201 25100933 PMC4106401

[B74] Mayr C. Stärke P. Partzsch J. Cederstroem L. Schüffny R. Shuai Y. (2012). Waveform driven plasticity in BiFeO3 memristive devices: model and implementation. Adv. Neural Inf. Process. Syst. 25, 1700–1708.

[B75] Merolla P. Arthur J. Akopyan F. Imam N. Manohar R. Modha D. S. (2011). A digital neurosynaptic core using embedded crossbar memory with 45pJ per spike in 45 nm, in Custom Integrative Circuits Conference (San Jose, CA), 1–4 10.1109/CICC.2011.6055294

[B76] Mu Y. Poo M. M. (2006). Spike timing-dependent LTP/LTD mediates visual experience-dependent plasticity in a developing retinotectal system. Neuron 50, 115–125. 10.1016/j.neuron.2006.03.009 16600860

[B77] Nessler B. Pfeiffer M. Buesing L. Maass W. (2013). Bayesian computation emerges in generic cortical microcircuits through spike-timing-dependent plasticity. PLoS Comput. Biol. 9:e1003037. 10.1371/journal.pcbi.1003037 23633941 PMC3636028

[B78] Noack M. Partzsch J. Mayr C. Schüffny R. (2010). Biology-derived synaptic dynamics and optimized system architecture for neuromorphic hardware, in 17th International Conference on Mixed Design of Integrated Circuits and Systems (Warsaw), 219–224.

[B79] Ohno T. Hasegawa T. Tsuruoka T. Terabe K. Gimzewski J. K. Aono M. (2011). Short term plasticity and long term potentiation mimicked in single inorganic synapses. Nat. Mater. 10, 591–595. 10.1038/nmat3054 21706012

[B80] Pershin Y. V. Di Ventra M. (2008). Spin memristive systems: spin memory effects in semiconductor spintronics. Phys. Rev. B 78:113309 10.1103/PhysRevB.78.113309

[B81] Querlioz D. Bichler O. Dollfus P. Gamrat C. (2013). Immunity to device variations in a spiking neural network with memristive nanodevices. IEEE Trans. Nanotechnol. 12, 288–295 10.1109/TNANO.2013.2250995

[B82] Querlioz D. Dollfus P. Bichler O. Gamrat C. (2011). Learning with memristive devices: how should we model their behavior? in 7th IEEE/ACM International Symposium on Nanoscale Architectures (San Diego, CA), 150–156.

[B83] Rangan V. Ghosh A. Aparin V. Cauwenberghs G. (2010). A subthreshold aVLSI implementation of the Izhikevich simple neuron model. Conf. Proc. IEEE Eng. Med. Biol. Soc. 2010, 4164–4167. 10.1109/IEMBS.2010.5627392 21096884

[B84] Shuai Y. Ou X. Luo W. Du N. Wu C. Zhang W. (2013). Nonvolatile multilevel resistive switching in Ar+ irradiated BiFeO3 thin films. IEEE Electron Device Lett. 34, 54–56 10.1109/LED.2012.2227666

[B85] Shuai Y. Zhou S. Wu C. Zhang W. Bürger D. Slesazeck S. (2011). Control of rectifying and resistive switching behavior in bifeo3 thin films. Appl. Phys. Express 4, 095802 10.1143/APEX.4.095802

[B86] Sjöström J. Gerstner W. (2010). Spike-timing dependent plasticity. Scholarpedia 5:1362 10.4249/scholarpedia.1362

[B87] Snider G. S. (2008). Spike-timing-dependent learning in memristive nanodevices, in IEEE International Symposium Nano Architecture (Anaheim, CA), 85–92 10.1109/NANOARCH.2008.4585796

[B88] Strukov D. B. Snider G. S. Stewart D. R. Williams R. S. (2008). The missing memristor found. Nature 453, 80–83. 10.1038/nature06932 18451858

[B89] Suri M. Querlioz D. Bichler O. Palma G. Vianello E. Vuillaume D. (2013). Bio-inspired stochastic computing using binary CBRAM synapses. IEEE Trans. Electron Devices 60, 2402–2409 10.1109/TED.2013.2263000

[B90] Upadhyaya H. M. Chandra S. (1995). Polarity dependent memory switching behavior in Ti/Cd Pb S/Ag system. Semicond. Sci. Technol. 10, 332–338 10.1088/0268-1242/10/3/016

[B92] Varela J. Sen K. Gibson J. Fost J. Abbott L. F. Nelson S. B. (1997). A quantitative description of short term plasticity at excitatory synapses in layer 2/3 of rat visual cortex. J. Neurosci. 17, 7926–7940. 9315911 10.1523/JNEUROSCI.17-20-07926.1997 PMC6793910

[B93] Vincent A. F. Locatelli N. Zhao W. S. Klein J.-O. Galdin-Retailleau S. Querlioz D. (2015). Analytical macrospin modeling of the stochastic switching time of spin transfer-torque magnetic tunnel junctions. IEEE Trans. Electron Devices 62, 164–170 10.1109/TED.2014.2372475

[B94] Vincent A. Larroque J. Zhao W. S. Ben Romdhane N. Bichler O. Gamrat C. (2014). Spin-transfer torque magnetic memory as a stochastic memristive synapse, in IEEE International Symposium Circuits System (Melbourne, VIC), 1074–1077 10.1109/ISCAS.2014.6865325

[B95] Wang X. Chen Y. Xi H. Li H. Dimitrov D. (2009). Spintronic memristor through spin-torque-induced magnetization motion. IEEE Electron Device Lett. 30, 294–297 10.1109/LED.2008.2012270

[B96] Waser R. Aono M. (2007). Nanoionics-based resistive switching memories. Nat. Mater. 6, 833–840. 10.1038/nmat2023 17972938

[B97] Wijekoon J. Dudek P. (2008). Compact silicon neuron circuit with spiking and bursting behavior. Neural Netw. 21, 524–534. 10.1016/j.neunet.2007.12.037 18262751

[B98] Wong H.-S. P. Lee H.-Y. Yu S. Chen Y.-S. Wu Y. Chen P.-S. (2012). Metal oxide RRAM. Proc. IEEE 100, 1951–1970 10.1109/JPROC.2012.2190369

[B99] Wu X. Zhou P. Li J. Chen L. Y. Lu H. B. Lin Y. Y. (2007). Reproducible unipolar resistance switching in stoichiometric ZrO2 films. Appl. Phys. Lett. 90, 183507 10.1063/1.2734900

[B100] Yamada H. Garcia V. Fusil S. Boyn S. Marinova M. Gloter A.. (2013). Giant electroresistance of super-tetragonal BiFeO3-based ferroelectric tunnel junctions. ACS Nano. 7, 5385–5390. 10.1021/nn401378t 23647323

[B101] Yang R. Terabe K. Yao Y. Tsuruoka T. Hasegawa T. Gimzewski J. K.. (2013). Synaptic plasticity and memory functions achieved in a WO3-x based nanoionics device by using the principle of atomic switch operation. Nanotechnology 24, 384003. 10.1088/0957-4484/24/38/384003 23999098

[B101a] You T. Du N. Slesazeck S. Mikolajick T. Li G. Bürger D.. (2014). Bipolar electric-field enhanced trapping and detrapping of mobile donors in BiFeO_3_ memristors. ACS Appl Mater Interfaces 6, 19758–19765. 10.1021/am504871g 25366867

[B102] Young J. M. (2007). Cortical reorganization consistent with spike timing-but not correlation-dependent plasticity. Nat. Neurosci. 10, 887–895. 10.1038/nn1913 17529985

[B103] Yu S. Gao B. Fang Z. Yu H. Kang J. Wong H.-S. P. (2013). Stochastic learning in oxide binary synaptic device for neuromorphic computing. Front. Neurosci. 7:186. 10.3389/fnins.2013.00186 24198752 PMC3813892

[B104] Zamarreño-Ramos C. Camuñas-Mesa L. A. Pérez-Carrasco J. A. Masquelier T. Serrano-Gotarredona T. Linares-Barranco B. (2011). On spike-timing-dependentplasticity, memristive devices, and building a self-learning visual cortex. Front. Neurosci. 5:26. 10.3389/fnins.2011.00026 21442012 PMC3062969

[B105] Zhang L. Tao H. Holt C. Harris W. Poo M. (1998). A critical window for cooperation and competition among developing retinotectal synapses. Nature 395, 37–44. 10.1038/25665 9738497

[B106] Zhang Y. Zhao W. Klein J.-O. Kang W. Querlioz D. Zhang Y. (2014). Spintronics for low-power computing, in Design, Automation and Test in Europe Conference and Exhibition (Dresden), 1–6 10.7873/DATE.2014.316

